# CTCF Regulates Erythroid Differentiation Through Control of Core Erythroid Transcription Factors

**DOI:** 10.3390/biom16040549

**Published:** 2026-04-08

**Authors:** Lorena García-Gaipo, Vanessa Junco, Lucía García-Gutiérrez, Verónica Torrano, Rosa Blanco, Alexandra Wiesinger, Rujula Pradeep, Jose Luis Arroyo, Ana Batlle-López, Javier León, Manuel Rosa-Garrido, M. Dolores Delgado

**Affiliations:** 1 Institute of Biomedicine and Biotechnology of Cantabria (IBBTEC), CSIC–University of Cantabria, 39011 Santander, Spain; lggaipo@gmail.com (L.G.-G.); vanessajunco1997@gmail.com (V.J.); lucia.garcia@unican.es (L.G.-G.); rosa.blanco@unican.es (R.B.); wiesingeralexandra@gmail.com (A.W.); abatllelopez@gmail.com (A.B.-L.); javier.leon@unican.es (J.L.); 2 Department of Molecular Biology, University of Cantabria, 39011 Santander, Spain; 3 Cell Cycle, Stem Cell Fate and Cancer Laboratory, Marqués de Valdecilla Research Institute (IDIVAL), 39011 Santander, Spain; 4 Department of Biochemistry and Molecular Biology, University of the Basque Country (UPV/EHU), 48940 Leioa, Spain; veronica.torrano@ehu.eus; 5 Biomedical Research Networking Center on Cancer (CIBERONC), 28029 Madrid, Spain; 6 Department of Data Science, The Fu Foundation School of Engineering and Applied Science, Columbia University, New York, NY 10027, USA; rp3248@columbia.edu; 7 Blood and Tissue Bank of Cantabria, Marqués de Valdecilla Foundation, Marqués de Valdecilla Research Institute (IDIVAL), 39011 Santander, Spain; arroyo.jl@fmvaldecilla.es; 8 Department of Hematology, Marqués de Valdecilla University Hospital–IDIVAL, 39011 Santander, Spain

**Keywords:** erythropoiesis, erythroid transcription factors, CTCF, *LMO2*, *KLF1*, *MYB*, *ETS1*

## Abstract

Erythropoiesis is tightly regulated by lineage-specific transcription factors that govern erythroid commitment, proliferation, and differentiation. A core erythroid transcriptional network, together with non-DNA-binding cofactors, occupies regulatory regions of genes essential for erythroid development. This process is further shaped by epigenetic mechanisms, including histone post-translational modifications and long-range chromatin interactions. CCCTC-binding factor (CTCF) is a multifunctional regulator with a central role in three-dimensional chromatin organization. Although CTCF has been implicated in hematopoietic differentiation and leukemogenesis, its specific function in erythropoiesis remains poorly defined. Here, we investigated the role of CTCF during erythroid differentiation using two complementary models: pluripotent K562 leukemia cells and primary human CD34^+^ hematopoietic stem/progenitor cells, each induced toward the erythroid lineage by distinct stimuli. In both systems, CTCF silencing impaired erythroid differentiation by repression of key erythroid transcription factor genes, including *LMO2*, *KLF1*, *MYB*, and *ETS1*. This repression was associated with enrichment of repressive histone marks at CTCF-binding sites within their regulatory regions. Moreover, CTCF cooperated with cohesin to establish and stabilize long-range chromatin interactions at these loci. These results provide new insight into how CTCF-dependent chromatin regulation contributes to normal erythroid development and suggest that perturbation of this regulatory axis may have implications for hematopoietic disorders and malignancies.

## 1. Introduction

Erythroid differentiation is a highly regulated, multistep process that progresses from hematopoietic stem cells to committed erythroid progenitors, including burst-forming unit erythroid (BFU-E) and colony-forming unit erythroid (CFU-E). These progenitors differentiate into proerythroblasts, which subsequently mature through basophilic, polychromatophilic, and orthochromatic erythroblast stages, ultimately undergoing enucleation to form reticulocytes and mature red blood cells [1]. Erythropoiesis is tightly controlled at multiple levels by cytokines, growth factors, and hormones, together with specific transcriptional regulators (for reviews, see [1,2,3]). Erythroid differentiation depends on the precisely timed expression of lineage-specific transcription factors that direct cell commitment, proliferation, and maturation. The core erythroid network (CEN) of transcription factors includes GATA1, TAL1 (SCL), and *KLF1* (EKLF), together with non-DNA-binding transcription cofactors such as LDB1 and *LMO2* [2,4,5]. These complexes, often in combination with additional transcription factors, occupy regulatory regions of genes involved in all aspects of erythropoiesis [6]. Accordingly, GATA1, TAL1 and *KLF1* are considered erythroid “master regulators” [6,7]. GATA1 is highly expressed in erythroid cells and, during erythroid differentiation, represses *GATA2* expression, a process known as “GATA switching” that is critical for proper erythropoiesis [6,8]. TAL1 directly binds to *LMO2*, forming key regulatory complexes, and *KLF1* expression is indispensable for the maturation of erythroblasts into erythrocytes and for the activation of *β-globin* gene expression during terminal erythroid differentiation [9]. Other transcription factors also play important roles in erythropoiesis. The proto-oncogene *MYB* is highly expressed in immature hematopoietic cells, and its expression declines during differentiation. *MYB* promotes erythropoiesis by transactivating *KLF1* and *LMO2* expression [10]. In contrast, members of the ETS transcription factor family, *ETS1* and FLI1, inhibit erythroid differentiation, in part through the downregulation of *GATA1* [11,12]. In addition, a transcriptional regulatory “heptad”—comprising TAL1, LYL1, *LMO2*, GATA2, FLI1, ERG, and RUNX1—has recently been identified as a key regulatory module that is frequently disrupted in hematopoietic malignancies [13,14].

Gene expression during erythroid differentiation is also regulated by epigenetic mechanisms, including histone post-translational modifications and long-range chromatin interactions [2,4,7,15]. CCCTC-binding factor (CTCF) is a multifunctional regulator with central roles in mediating three-dimensional chromatin organization. Its functions include transcriptional regulation, insulation, chromatin looping, boundary definition, and epigenetic control of target genes (for recent reviews, see [16,17,18] and references therein). CTCF plays critical roles in processes such as cell proliferation, differentiation and DNA damage repair [19,20]. Its involvement in cancer, including hematological malignancies, has been extensively investigated [21,22,23,24]. Our group was the first to describe differentiation-dependent changes in CTCF expression and post-translational modification during myeloid cell differentiation [25]. CTCF overexpression in pluripotent K562 cells drives differentiation towards the erythroid lineage [26]. CTCF also plays essential roles in regulating hematopoietic stem cell differentiation [27,28]. Dynamic CTCF-binding sites that are critical for hematopoiesis have been identified [29] and reported interactions between CTCF and LDB1 enable the activation of multiple erythroid genes through CTCF-mediated enhancer looping [30]. Recently, CTCF has been shown to be selectively required for maintaining chromatin accessibility during erythropoiesis [31]. However, it remains unclear whether CTCF has a direct impact on the erythroid transcriptional network.

Despite the growing body of evidence linking CTCF to erythropoiesis, its precise role in this process remains incompletely understood. In this work, we provide further insights into CTCF function during erythroid differentiation induced by cytosine arabinoside (Ara-C) or imatinib in K562 cells, and by erythropoietin in primary human CD34^+^ hematopoietic stem/progenitor cells. Upon CTCF downregulation in these models, we demonstrate that CTCF is essential for erythroid differentiation, an effect mediated through the regulation of key erythroid transcription factors such as *KLF1* and *LMO2*, among others.

## 2. Materials and Methods

### 2.1. Cell Cultures and Differentiation

The K562 cell line (obtained from American Type Culture Collection; Manassas, VA, USA) is derived from a human chronic myeloid leukemia. KCTCF-D11 and KpCDNA are K562 cells stably transfected with pCDNA-CTCF vector or the empty vector, respectively [26]. Cells were grown in RPMI-1640 medium supplemented with 10% fetal bovine serum (Lonza; Basel, Switzerland), 150 µg/mL gentamicin and 2 µg/mL ciprofloxacin. Exponentially growing K562 cells were treated with 1 μM 1-β-D-arabinofuranosylcytosine (Ara-C) (Sigma-Aldrich; Burlington, MA, USA) or 0.5 or 1 μM Imatinib (LC Laboratories; Woburn, MA, USA) for up to 5 days to induce erythroid differentiation [26,32]. Differentiation was assessed by scoring hemoglobin-containing cells after benzidine staining. The benzidine test is based on the pseudoperoxidase activity of hemoglobin and is widely used as a functional readout of hemoglobinized erythroid cells [33]. A minimum of 200 cells were counted using the ImageJ software (version 1.54j) and the number of hemoglobin-producing cells (blue) relative to non-hemoglobin producing cells (white) was determined and expressed as percentage of benzidine-positive cells. Cell viability was assessed by the dye exclusion test with Trypan Blue (Sigma-Aldrich). The cleavage of poly(ADP-ribose)polymerase-1 (PARP1) indicative of apoptosis, was analyzed by Western blot, as described below.

Primary CD34^+^ cells were obtained from human umbilical cord blood kindly donated by the Blood and Tissue Bank of Cantabria with approval from the local ethics committee (Ethics Committee on Clinical Research of Cantabria; Santander, Spain). Cord blood donations were obtained after written informed consent for research purposes. Samples were anonymized by the Blood and Tissue Bank of Cantabria prior to analysis, ensuring donor non-identifiability. CD34^+^ cells were obtained from at least 80 independent cord blood donors. Mononuclear cells were isolated by density gradient centrifugation on Ficoll-Paque Plus (GE Healthcare; Chicago, IL, USA). CD34^+^ cells were purified using a magnetic beads separation system (CD34 MicroBead Kit Ultrapure and MACS Columns, Miltenyi Biotec; Bergisch Gladbach, Germany) according to the manufacturer’s instructions. CD34^+^ cells were cultured in StemSpanTM SFEM II medium supplemented with StemSpamTM CD34^+^ Expansion Supplement 10X containing Flt3L, SCF, IL-3, IL-6 and TPO (StemCell Technologies; Vancouver, BC, Canadá) for up to 3 days. The purity of isolated CD34^+^ cells was assessed by flow cytometry using CD34-PE antibody (Miltenyi Biotec) on a FACScan cytometer (BD Biosciences; Franklin Lakes, NJ, USA). Expanded CD34^+^ cells were treated with 3 or 6 U/mL Erythropoietin (EPO) (R&D Systems; Minneapolis, MN, USA) for up to 14 days to induce erythroid differentiation. Cells were collected at different time points, and erythroid differentiation was analyzed by flow cytometry for the expression of the erythroid-specific marker Glycophorin-A (CD235a) using the anti-CD235a VioBlue antibody (Miltenyi Biotec) on a BD FACSDiva flow cytometer, following standard procedures. Benzidine staining was also performed in parallel.

### 2.2. Lentiviral Production and Infection

For lentivirus production, HEK293T cells were transfected using PEI with the lentiviral packaging plasmids (pCMV-VSV-G and psPAX2 from Addgene; Watertown, MA, USA) and the construct of interest as previously described [34]. The following transfer plasmids were used: pLKO.1 puro empty vector and pLKO shCTCF TRCN0000321371 (Sigma-Aldrich); pTRIPZ empty vector and pTRIPZ Human CTCF shRNA V3THS_409881 (DharmaconTM, GE Healthcare). Supernatants were collected 48 h post-transfection, and lentiviral particles were precipitated using PEG8000 (Sigma-Aldrich) Viral titers were estimated using HeLa cells. K562 cells were transduced at a multiplicity of infection (MOI) ≥ 3 in serum-free medium containing 5 μg/mL polybrene for 12 h. Complete medium was then added. At 36 h post-transduction, cells were washed to remove residual lentiviral particles and transferred to fresh medium containing puromycin (1 μg/mL), to eliminate most uninfected cells.

For CD34^+^ cell transduction, a MOI of 3 was used. To enhance transduction efficiency, cells were incubated with retronectin. The appropriate volume of lentiviral suspension was added and incubated for 5 h at 37 °C. Lentivirus was then removed, and cells were resuspended in serum-free medium. After 12 h, StemSpamTM SFEM II supplemented with StemSpanTM CD34^+^ Expansion Supplement (StemCell Technologies; Vancouver, BC, Canadá) was added to reach the appropriate total volume. At 48 h post-infection, the medium was replaced with new medium containing puromycin (0.5 μg/mL) to select transduced cells.

### 2.3. RNA Extraction and RT-qPCR Analysis

For RT-qPCR, total RNA was isolated using either the TRI Reagent^®^ Solution (Invitrogen, ThermoFisher Scientific; Waltham, MA, USA) or the RNeasy Mini Kit (Qiagen; Venlo, Netherlands). cDNA was synthesized by reverse transcription (RT) using the iScript cDNA Synthesis Kit (Bio-Rad; Hercules, CA, USA). Quantitative polymerase chain reaction (qPCR) was performed with specific primers (see Supplementary Table S1) using the iTaq™ Universal SYBR^®^ Green Supermix (Bio-Rad) and a CFX Connect Real-Time PCR Detection System (Bio-Rad). mRNA levels were normalized against RPS14 (ribosomal protein S14) levels as previously described [34].

### 2.4. Gene Expression Profiling

Total RNA was prepared using RNeasy kit (Qiagen). Biotinylated cRNA was obtained from RNA samples from KpCDNA and KCTCF-D11 cells and hybridized to Affymetrix HG-U133A Plus 2.0 chip in the Genomic Facility of Cancer Research Center (Salamanca, Spain). Raw data were obtained as CEL files and processed using dChip (DNA-Chip Analyzer) version 1.3. Probe-level intensities were summarized using the HG-U133 Plus 2.0 CDF file, and probe sets were annotated using the corresponding gene information file. Arrays were normalized across samples using invariant set normalization, and expression values were calculated using the model-based expression index, which reduces the influence of outlier probes. Normalized and modeled expression values were used for all subsequent analyses. Differential expression analysis was performed using the Compare Samples function in dChip. Control KpCDNA cells were used as the baseline group, and CTCF-overexpressing KCTCF-D11 cells were used as the experimental group. Differentially expressed genes were identified based on fold-change criteria and minimum expression difference thresholds as implemented in dChip. Genes passing the dChip filtering criteria were classified as differentially expressed, corresponding to cases in which the confidence interval of the fold change excluded 1. The resulting list of differentially expressed genes was exported for downstream analysis. Data are available in the Gene Expression Omnibus database; accession number GSE319471. Gene Ontology (GO) enrichment analysis was performed using the PANTHER classification system. The results were visualized using GOplot.

### 2.5. Western Blot Analysis

Cells were lysed in RIPA lysis buffer and immunoblots were performed as previously described [34]. For histone extraction, cells were harvested, washed twice with ice-cold PBS, and lysed in Triton Extraction Buffer (PBS with 0.5% Triton X-100, 300 mM NaCl, and protease/phosphatase inhibitors) for 10 min on ice. After centrifugation (2000 rpm, 10 min, 4 °C), the supernatant was discarded and the pellet was washed once more with TEB. Histones were extracted by resuspending the pellet in 0.2 N HCl and incubating overnight at 4 °C with agitation. Following centrifugation (10,000 rpm, 10 min, 4 °C), the supernatant containing histones was collected and neutralized with Tris (pH 8.8). Samples were resolved by SDS-PAGE, and Coomassie staining was used to verify equal histone loading. The antibodies used are listed in Supplementary Table S2. Blots were developed with secondary antibodies conjugated to IRDye680 or IRDye800 (Li-Cor Biosciences; Lincoln, NE, USA) (Supplementary Table S3) and immunocomplexes were detected using an Odyssey infrared-imaging system (Li-Cor Biosciences). Densitometric analysis of immunoblots was performed using ImageJ software version 1.54k for Windows). Protein expression was normalized to the loading control (actin or tubulin levels) and presented as relative values. Original Western Blot images including molecular weight markers and positive controls are available in Supplementary Materials (Figure S8).

### 2.6. Chromatin Immunoprecipitation (ChIP)

ChIP assays were performed using the Pierce Magnetic ChIP Kit (Thermo Fisher Scientific; Waltham, MA, USA) as previously described [35]. Cells were fixed in formaldehyde, lysed, treated with micrococcal nuclease and sonicated using a Bioruptor UCD-200TM (Diagenode, Liège, Belgium). ChIP was performed using ChIP-Grade Protein A/G Magnetic Beads coupled to different antibodies: anti-CTCF-Ab-1 (07-729) from Millipore (Burlington, MA, USA), anti-CTCF (ab10571) from Abcam (Cambridge, UK); anti-CTCF (612149) from BD Biosciences (Franklin Lakes, NJ, USA) and anti-H3K27me3 (07-449) from Millipore (Supplementary Table S4). Real-time PCR of immunoprecipitated DNA was performed in duplicate with equal amounts of specific antibody-immunoprecipitated sample, bead-only control, and input. Primers used for ChIP assays, corresponding to CTCF binding sites in erythroid genes, are listed in Supplementary Table S5. Positive (H42.1 rDNA) and negative (H4 rDNA) controls for CTCF binding were included [35]. The comparative cycle threshold method was used for data analysis as described [35]. Signals were normalized to input, and fold enrichment was calculated relative to the no-antibody control.

### 2.7. Bioinformatic Analysis

Data from the Encyclopedia of DNA Elements (ENCODE) consortium were used to predict and analyze potential CTCF-binding sites (CTSs) in erythroid genes. All analyses were performed using the GRCh38 (hg38) assembly of the human genome. CTCF ChIP-seq peak files for the K562 cell line were retrieved from the ENCODE database (GEO accession: GSM935407) and used to identify CTSs, which subsequently guided primer design for chromatin immunoprecipitation (ChIP) assays to validate CTCF binding. ChIP-seq datasets from additional cell lines were analyzed to assess conservation of CTCF binding, including lymphoblastoid GM12878 cells (GEO: GSM935611), lung adenocarcinoma A549 cells (GEO: GSE92808), lung fibroblasts IMR90 (GEO: GSM935404), and neuroblastoma SK-N-SH cells (GEO: GSM1003633). Cohesin occupancy was evaluated using RAD21 ChIP-seq data generated in K562 cells and retrieved from ENCODE (GEO: GSE231244). CTCF-mediated chromatin interactions were examined using CTCF ChIA-PET data from K562 cells available through ENCODE (GEO: GSE177471). In addition, genome-wide chromatin contact profiles were analyzed using intact Hi-C data generated in K562 cells as part of the ENCODE project (aggregate series ENCSR36ZWJ). Significant chromatin interactions were identified from the Hi-C data using the FitHiC [36,37] algorithm under NASA (non-assumption of specific architecture) conditions, allowing unbiased detection of statistically significant contact pairs.

### 2.8. Statistical Analysis

Statistical analyses were performed using GraphPad Prism version 10.6.1 for Windows, (GraphPad Software, Boston, MA, USA). The specific analysis used to assess the statistical significance of each experiment is specified in the corresponding figure legend. Bars represent the mean ± standard deviation (SD) and “*n*” values correspond to the number of independent experiments included. GraphPad style (GP) was used to represent *p* values: * *p* < 0.05; ** *p* < 0.01; *** *p* < 0.001; **** *p* < 0.0001. *p* values ≥ 0.05 were considered not significant.

## 3. Results

### 3.1. CTCF Downregulation Inhibits the Erythroid Differentiation in K562 Cells

**Figure 1:**
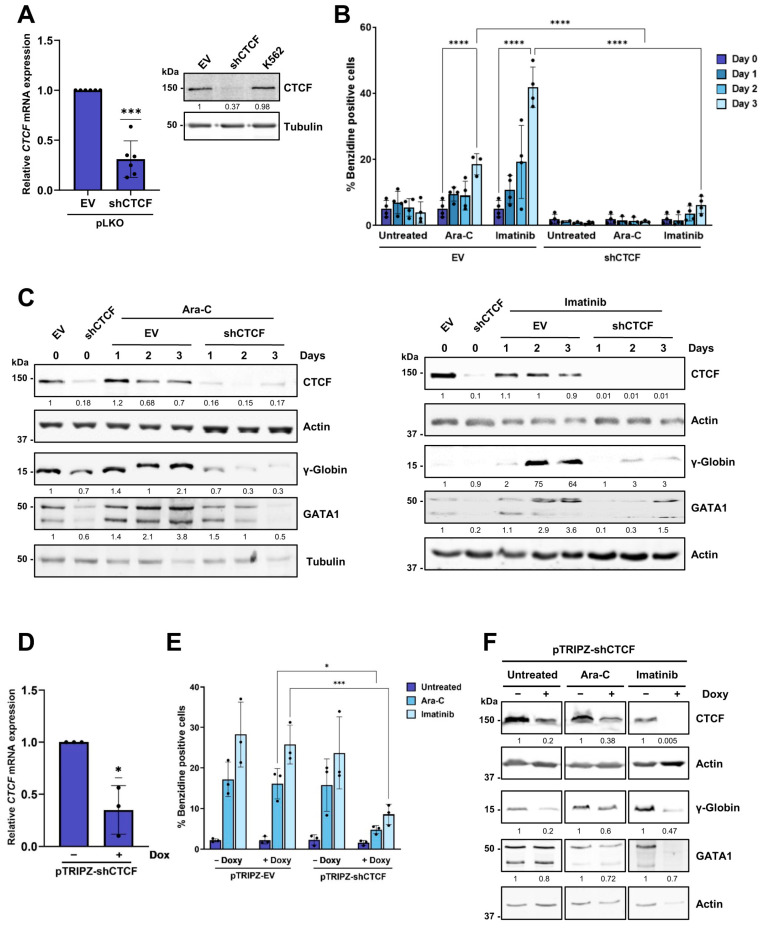
Constitutive and inducible CTCF downregulation inhibits erythroid differentiation in K562 cells. (**A**) *CTCF* expression was analyzed by RT-qPCR in K562 cells following infection with pLKO empty vector (EV) or pLKO shCTCF lentiviruses and puromycin selection for two days. Expression levels were normalized to *RPS14*. Data represent mean ± SD (*n* = 6). *** *p* < 0.001 by two-tailed one-sample *t*-test. CTCF protein levels were analyzed by Western blot in K562 infected cells. Protein signal quantification was normalized to the loading control (tubulin). (**B**) Benzidine staining of K562 cells infected with pLKO empty vector (EV) or pLKO shCTCF and treated with 1 µM Ara-C or 0.5 µM imatinib for 3 days. In each experiment, a minimum of 200 cells were counted, and the percentage of benzidine-positive cells is shown. Data represent mean ± SD (*n* = 3). **** *p* < 0.0001 by two-way ANOVA followed by Tukey’s post hoc multiple-comparisons test. (**C**) Protein expression of CTCF, γ-globin, and GATA1 was analyzed by Western blot in K562 cells following infection with pLKO empty vector (EV) or pLKO shCTCF and treatment with 1 µM Ara-C or 0.5 µM imatinib for 3 days. Protein signal quantification was normalized to the loading control (actin or tubulin). (**D**) *CTCF* expression was analyzed by RT-qPCR in K562 cells following infection with inducible pTRIPZ shCTCF lentiviruses, two days of puromycin selection, and three days of induction with 2 µg/mL doxycycline. Expression levels were normalized to RPS14. Data represent mean ± SD (*n* = 3). * *p* < 0.05 by two-tailed one-sample *t*-test. (**E**) Benzidine staining of K562 cells infected with inducible pTRIPZ empty vector (EV) or pTRIPZ shCTCF, as described in (**D**), and treated with 1 µM Ara-C or 0.5 µM imatinib for 3 days. In each experiment, a minimum of 200 cells were counted, and the percentage of benzidine-positive cells is shown. Data represent mean ± SD (*n* = 3). * *p* < 0.05; *** *p* < 0.001 by two-way ANOVA. (**F**) Protein expression of CTCF, γ-globin and GATA1 was analyzed by Western blot in K562 cells following infection with inducible pTRIPZ shCTCF, as described in (**D**), and treatment with Ara-C or imatinib for 3 days. Protein signal quantification was normalized to the loading control (actin). All original Western blot images can be found in the Supplementary Materials.

To model erythroid differentiation, we employed the human myeloid leukemia K562 cell line. K562 cells are capable of differentiating into the erythroid lineage in response to drugs such as Ara-C or imatinib. Exponentially growing cells were treated with either 1 µM Ara-C or 1 µM imatinib for up to 5 days. As previously described [26,32], both drugs induced cell growth arrest and erythroid differentiation, as assessed by the benzidine staining of hemoglobinized cells and the upregulation of erythroid markers including glycophorin A and γ-globin (Supplementary Figure S1A–D). To determine the effect of *CTCF* downregulation on the erythroid differentiation, we generated lentiviral particles expressing a short-hairpin RNA targeting *CTCF* (shCTCF) or the control pLKO vector. K562 cells were infected with shCTCF or the empty vector (pLKO) and selected with puromycin to eliminate the uninfected cells. The shCTCF construct effectively reduced *CTCF* mRNA and protein levels (Figure 1A). Erythroid differentiation was then induced by Ara-C or imatinib. Differentiation was first assessed by scoring hemoglobin-producing cells with the benzidine test. Control K562 vector-infected cells showed a marked increase in benzidine-positive cells upon treatment with either Ara-C or imatinib (Figure 1B). In contrast, CTCF knockdown dramatically impaired the accumulation of hemoglobinized cells, both in untreated conditions and after drug induction. To verify these observations, we measured the expression of erythroid markers, γ-globin and GATA1, by Western blot. As expected, CTCF protein levels were clearly reduced in shCTCF-infected cells, with or without Ara-C (Figure 1C, left panel) or imatinib (Figure 1C, right panel). In control cells, *γ-globin* and *GATA1* expression increased upon drug-induced differentiation. However, this upregulation was abrogated in CTCF-depleted cells (Figure 1C).

To further validate these results, we used a lentiviral construct (pTRIPZ) for doxycycline-inducible expression of the CTCF shRNA. K562 cells were infected and underwent selection with puromycin, followed by doxycycline administration for 3 days to induce shCTCF expression. The transduction efficiency was approximately 85%, as assessed by the expression of the fluorescent marker RFP. Induction of shCTCF resulted in a significant reduction in *CTCF* mRNA levels (Figure 1D). K562 cells, infected with either the pTRIPZ empty vector or pTRIPZ-shCTCF, were treated with Ara-C or imatinib, for induction of erythroid differentiation. The percentage of benzidine-positive cells increased following treatment with Ara-C or imatinib in cells infected with the empty vector, irrespective of doxycycline induction (Figure 1E). A similar increase in the benzidine-positive fraction was also observed in cells infected with pTRIPZ-shCTCF in the absence of doxycycline. However, doxycycline-induced downregulation of CTCF markedly reduced the number of hemoglobinized cells following treatment with Ara-C and imatinib (Figure 1E). Western blot analysis confirmed that the reduction in CTCF protein levels following doxycycline treatment in differentiating cells was associated with a decrease in the erythroid markers *γ-globin* and *GATA1* (Figure 1F).

To assess whether the impaired erythroid differentiation observed upon CTCF depletion could be attributed to reduced cell viability, we evaluated cell viability during the differentiation process in K562 cells. Trypan blue exclusion assays showed that CTCF knockdown did not result in a major loss of cell viability under these conditions (Supplementary Figure S2A). In addition, analysis of PARP1 cleavage by Western blot (Supplementary Figure S2B) did not reveal a significant increase in apoptosis upon CTCF depletion, indicating that the observed effects are not primarily due to a general reduction in cellular viability.

Altogether, these results demonstrate that, in both constitutive and doxycycline-inducible systems for CTCF knockdown, erythroid differentiation is significantly impaired when CTCF levels are reduced.

### 3.2. CTCF Downregulation Inhibits the Erythroid Differentiation in Primary CD34^+^ Stem/Progenitor Cells

To gain deeper insight into the role of CTCF during erythropoiesis, we used primary hematopoietic cells as a more physiologically relevant model. Human CD34^+^ hematopoietic stem/progenitor cells were isolated from cord blood and erythroid differentiation was induced with erythropoietin (EPO). To optimize experimental conditions, we tested two EPO concentrations (3 and 6 U/mL), assessing erythroid differentiation through the benzidine staining assay and expression of key erythroid marker genes. Following 5, 7 and 10 days of treatment, the proportion of benzidine-positive cells increased comparably across both EPO concentrations (Supplementary Figure S3A). As expected, the percentage of glycophorin A-positive cells (Supplementary Figure S3B) and γ-globin and GATA1 protein expression (Supplementary Figure S3C) increased upon EPO stimulation, further confirming erythroid lineage commitment.

**Figure 2:**
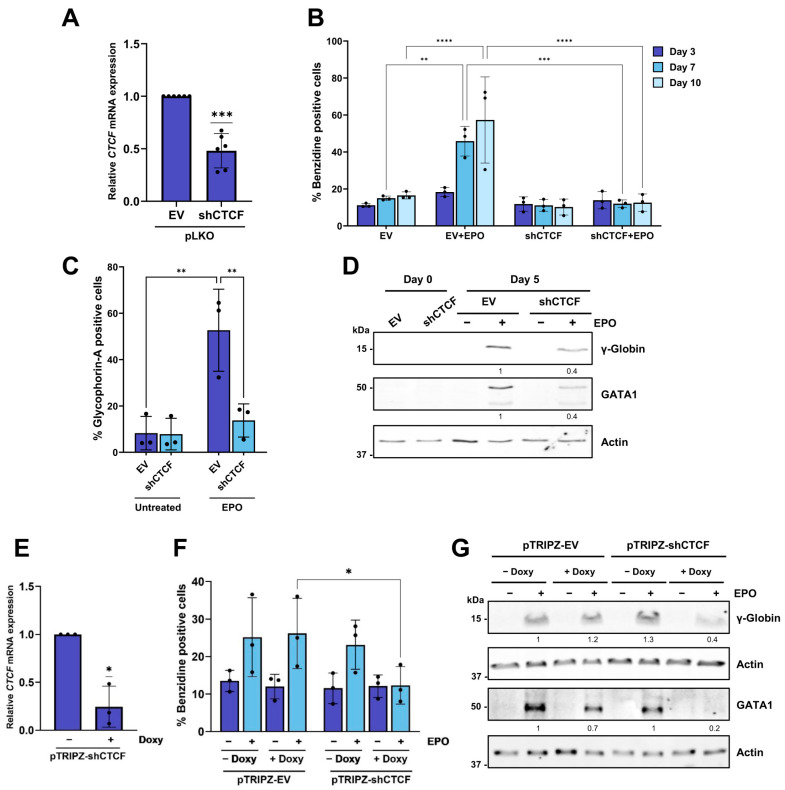
Constitutive and inducible CTCF downregulation inhibits erythroid differentiation in CD34^+^ cells. (**A**) *CTCF* expression was analyzed by RT-qPCR in CD34^+^ cells following infection with pLKO empty vector (EV) or pLKO shCTCF lentiviruses and puromycin selection for two days. Expression levels were normalized to *RPS14*. Data represent mean ± SD (*n* = 6). *** *p* < 0.001 by two-tailed one-sample *t*-test. (**B**) Benzidine staining of CD34^+^ cells infected with pLKO empty vector (EV) or pLKO shCTCF and treated with 3 U/mL erythropoietin (EPO) for 10 days. In each experiment, a minimum of 200 cells were counted, and the percentage of benzidine-positive cells is shown. Data represent mean ± SD (*n* = 3). ** *p* < 0.01, *** *p* < 0.001, **** *p* < 0.0001 by two-way ANOVA followed by Tukey’s post hoc multiple-comparisons test. (**C**) Percentage of glycophorin A-positive cells analyzed by flow cytometry after infection of CD34^+^ cells with pLKO empty vector (EV) or pLKO shCTCF and treatment with 3 U/mL erythropoietin (EPO) for 5 days. Data represent mean ± SD (*n* = 3). ** *p* < 0.01 by two-way ANOVA. (**D**) Protein expression of γ-globin and GATA1 was analyzed by Western blot in CD34^+^ cells following infection with pLKO empty vector (EV) or pLKO shCTCF and treatment with 3 U/mL erythropoietin (EPO) for 5 days. Protein signal quantification was normalized to the loading control (actin). (**E**) *CTCF* expression was analyzed by RT-qPCR in CD34^+^ cells following infection with inducible pTRIPZ shCTCF lentiviruses, two days of puromycin selection, and two days of induction with 2 µg/mL doxycycline. Expression levels were normalized to RPS14. Data represent mean ± SD (*n* = 3). * *p* < 0.05 by two-tailed one-sample *t*-test. (**F**) Benzidine staining of CD34^+^ cells infected with inducible pTRIPZ empty vector (EV) or pTRIPZ shCTCF, as described in (**E**), and treated with 3 U/mL erythropoietin (EPO) for 5 days. In each experiment, a minimum of 200 cells were counted, and the percentage of benzidine-positive cells is shown. Data represent mean ± SD (*n* = 3). * *p* < 0.05 by two-way ANOVA. (**G**) Protein expression of γ-globin and GATA1 was analyzed by Western blot in CD34^+^ cells following infection with inducible pTRIPZ empty vector (EV) or pTRIPZ shCTCF, as described in (**E**), and treatment with erythropoietin (EPO) for 5 days. Protein signal quantification was normalized to the loading control (actin). All original Western blot images can be found in the Supplementary Materials.

We then examined whether CTCF levels influence erythroid differentiation of CD34^+^ cells. To assess this, we silenced CTCF expression using both the constitutive and inducible lentiviral shRNA systems, as described above. Efficient downregulation of *CTCF* mRNA in CD34^+^ cells infected with pLKO shCTCF lentiviral particles was achieved (Figure 2A). Cells were then cultured in the presence of EPO for up to 10 days, and erythroid differentiation was evaluated using the benzidine staining assay. In cells infected with the empty vector, the proportion of hemoglobin-producing (benzidine-positive) cells progressively increased during EPO treatment (Figure 2B). In contrast, CTCF-depleted cells failed to show this increase, with benzidine-positive cell percentages remaining comparable between untreated and EPO-treated conditions (Figure 2B). Supporting these results, the percentage of glycophorin A-positive cells (Figure 2C), as well as γ-globin and GATA1 protein levels (Figure 2D), were markedly reduced in shCTCF-infected CD34^+^ cells compared with controls. Trypan blue exclusion assays showed that CTCF knockdown did not result in a major loss of cell viability (Supplementary Figure S2C).

To further characterize erythroid maturation, we analyzed forward scatter (FSC-A; cell size) and side scatter (SSC-A; cellular complexity) parameters within the glycophorin A-positive population (Supplementary Figure S4). EV-infected cells treated with EPO displayed reduced cell size and complexity, consistent with erythroid maturation. In contrast, shCTCF-infected cells treated with EPO exhibited FSC-A and SSC-A profiles comparable to untreated controls, indicating retention of a more immature phenotype and supporting impaired erythroid maturation upon CTCF depletion.

Finally, CD34^+^ cells were transduced with the inducible lentiviral pTRIPZ-shCTCF or the corresponding empty vector. Doxycycline-induced CTCF knockdown was confirmed (Figure 2E), and its impact on erythroid differentiation was evaluated using the benzidine staining assay (Figure 2F) and by measuring γ-globin and GATA1 expression (Figure 2G). CTCF downregulation resulted in a marked inhibition of erythroid differentiation in primary CD34^+^ stem/progenitor cells.

### 3.3. CTCF Downregulation Inhibits Erythroid Transcription Factor Expression

**Figure 3:**
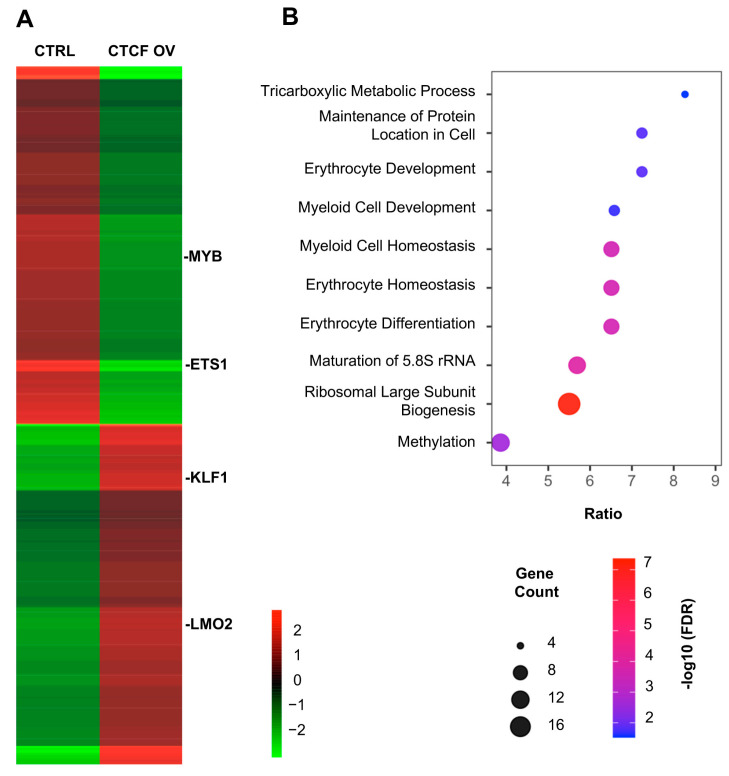
Global transcriptional changes induced by CTCF overexpression in K562 cells. (**A**) Heatmap showing differentially expressed genes following CTCF overexpression compared with control cells, represented as log_2_ fold change values. Red and green indicate relative upregulation and downregulation, respectively. Unsupervised hierarchical clustering reveals coordinated transcriptional responses, including key erythroid-associated genes. (**B**) Enriched categories are predominantly associated with erythroid differentiation and lineage-specific transcriptional regulation, consistent with activation of erythroid transcriptional programs upon CTCF overexpression. The x-axis represents the enrichment ratio (gene ratio). Dot size corresponds to the number of genes associated with each GO term, and dot color indicates statistical significance expressed as −log10(FDR).

Our functional analyses demonstrate that CTCF silencing severely impairs erythroid differentiation, indicating that CTCF is required for proper activation of the erythroid program. However, the pronounced arrest of differentiation observed upon CTCF downregulation limits the ability to capture early and potentially direct transcriptional effects of CTCF loss. To complement these loss-of-function studies and to define erythroid gene expression programs that are responsive to CTCF levels, we therefore performed transcriptomic profiling following CTCF overexpression. Consistent with our previous findings showing that CTCF overexpression in K562 cells enhances erythroid differentiation [26], transcriptomic analysis revealed widespread changes in gene expression upon increased CTCF levels. Unsupervised hierarchical clustering of differentially expressed genes revealed coordinated transcriptional responses, with distinct gene clusters showing either increased or decreased expression relative to control cells. These clusters included key transcription factors such as *LMO2*, *KLF1*, *MYB*, and *ETS1* (Figure 3A). Gene ontology analysis of differentially expressed genes revealed significant enrichment for biological processes associated with erythroid differentiation and lineage-specific transcriptional regulation (Figure 3B). Notably, these enriched categories were highly interconnected and reflected coordinated activation of erythroid-specific gene expression programs. Together, these results indicate that elevated CTCF levels are associated with activation of erythroid gene expression programs and suggest that key regulators of erythroid differentiation are responsive to CTCF levels.

**Figure 4:**
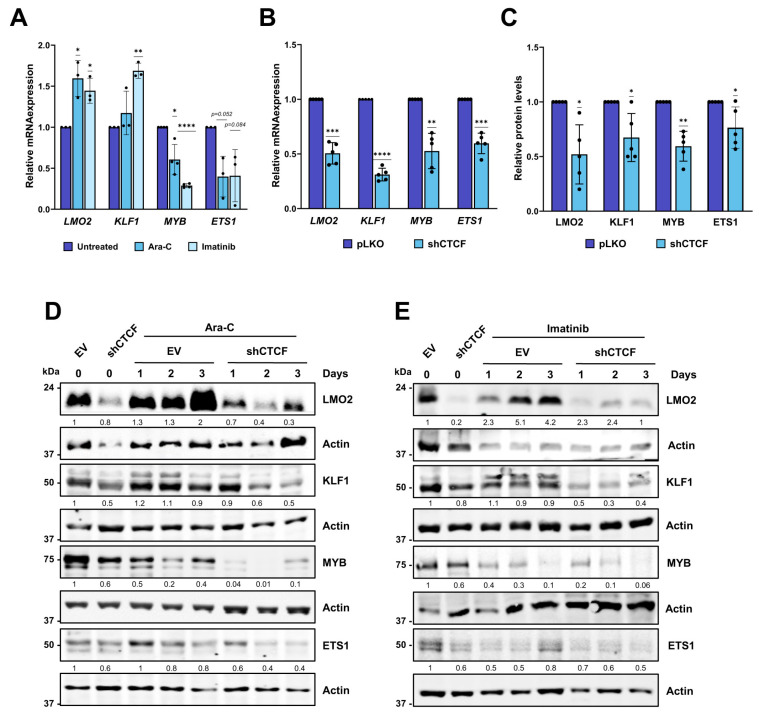
Effect of CTCF downregulation on erythroid genes expression upon differentiation. (**A**) mRNA expression levels of *LMO2*, *KLF1*, *MYB*, and *ETS1* were analyzed by RT-qPCR in K562 cells following treatment with 1 µM Ara-C for 72 h or 0.5 µM imatinib for 48 h. Expression levels were normalized to RPS14. Data represent mean ± SD (*n* ≥ 3). * *p* < 0.05, ** *p* < 0.01, **** *p* < 0.0001 by two-tailed one-sample *t*-test. (**B**) mRNA expression levels of *LMO2*, *KLF1*, *MYB*, and *ETS1* were analyzed by RT-qPCR after infection of K562 cells with pLKO (EV) or pLKO shCTCF and selection with puromycin for two days. Expression levels were normalized to RPS14. Data represent mean ± SD (n ≥ 3). ** *p* < 0.01, *** *p* < 0.001, **** *p* < 0.0001 by two-tailed one-sample *t*-test. (**C**) Densitometric quantification of *LMO2*, *KLF1*, *MYB*, and *ETS1* protein levels analyzed by Western blot in K562 cells following infection with pLKO empty vector (EV) or pLKO shCTCF, as described in (**B**). Data represent mean ± SD (*n* = 5). * *p* < 0.05, ** *p* < 0.01 by two-tailed one-sample *t*-test. (**D**,**E**) Protein expression of *LMO2*, *KLF1*, *MYB*, and *ETS1* was analyzed by Western blot in K562 cells following infection with pLKO empty vector (EV) or pLKO shCTCF and treatment with 1 µM Ara-C (**D**) or 0.5 µM imatinib (**E**) for 3 days. Protein signal quantification was normalized to the loading control (actin or tubulin). Quantification data from three independent Western blot experiments are shown in Supplementary Figure S5. The actin blot corresponding to *MYB* expression after Ara-C treatment is the same as that shown in Figure 1C. All original Western blot images can be found in the Supplementary Materials.

To further investigate the regulation of these transcription factors, we examined their mRNA expression following induction of erythroid differentiation with Ara-C or imatinib (Figure 4A). Upon differentiation, *LMO2* and *KLF1* expression was significantly upregulated, whereas *MYB* and *ETS1* expression was downregulated, consistent with their established roles in erythroid lineage specification. Importantly, CTCF knockdown in undifferentiated K562 cells resulted in a marked reduction in both mRNA (Figure 4B) and protein (Figure 4C) levels of these transcription factors, suggesting that CTCF is required to maintain their basal expression. To assess the impact of CTCF depletion during erythroid differentiation, K562 cells were transduced with either pLKO shCTCF or the empty vector control (pLKO) and treated with Ara-C or imatinib for up to three days. Protein expression was analyzed by Western blotting. *LMO2*, a cofactor essential to the core erythroid transcriptional network [6], was upregulated following differentiation with either agent (Figure 4D,E). In contrast, *LMO2* expression was markedly reduced in shCTCF-expressing cells at baseline (day 0) and remained suppressed throughout the differentiation time course (days 1–3). *KLF1*, another master regulator of erythroid differentiation and β-globin gene activation [9], displayed a similar pattern: its expression increased upon Ara-C or imatinib treatment in control cells but failed to be induced in CTCF-depleted cells (Figure 4D,E). Conversely, *MYB* and *ETS1*, which must be downregulated to permit erythroid differentiation [10,11], showed reduced expression during pharmacologically induced differentiation in control cells; this downregulation was even more pronounced in CTCF-silenced cells.

Taken together, these results demonstrate that CTCF depletion disrupts the proper expression of key erythroid transcription factors both at baseline and during induced differentiation, underscoring the critical role of CTCF in regulating erythroid transcriptional programs.

### 3.4. CTCF Knockdown Induces the Incorporation of Repressive Histone Marks at Regulatory Regions of Erythroid Transcription Factor Genes

We next investigated whether CTCF directly regulates the expression of *LMO2*, *KLF1*, *MYB*, and *ETS1* by binding to their regulatory regions. To identify potential CTCF-binding sites (CTSs) associated with these erythroid genes, we analyzed publicly available CTCF ChIP-seq datasets from the ENCODE project across five human cell lines: the erythroid K562 line, lymphoblastoid GM12878 cells, lung adenocarcinoma A549 cells, lung fibroblasts IMR90, and neuroblastoma SK-N-SH cells. To validate these in silico predictions, we performed chromatin immunoprecipitation assays to assess CTCF binding, as well as the occupancy of the repressive histone mark H3K27me3 following CTCF knockdown. In addition, ENCODE datasets for RAD21 (a cohesin subunit), CTCF ChIA-PET, and Hi-C in K562 cells were analyzed to evaluate the potential involvement of these CTSs in long-range chromatin interactions.

**Figure 5:**
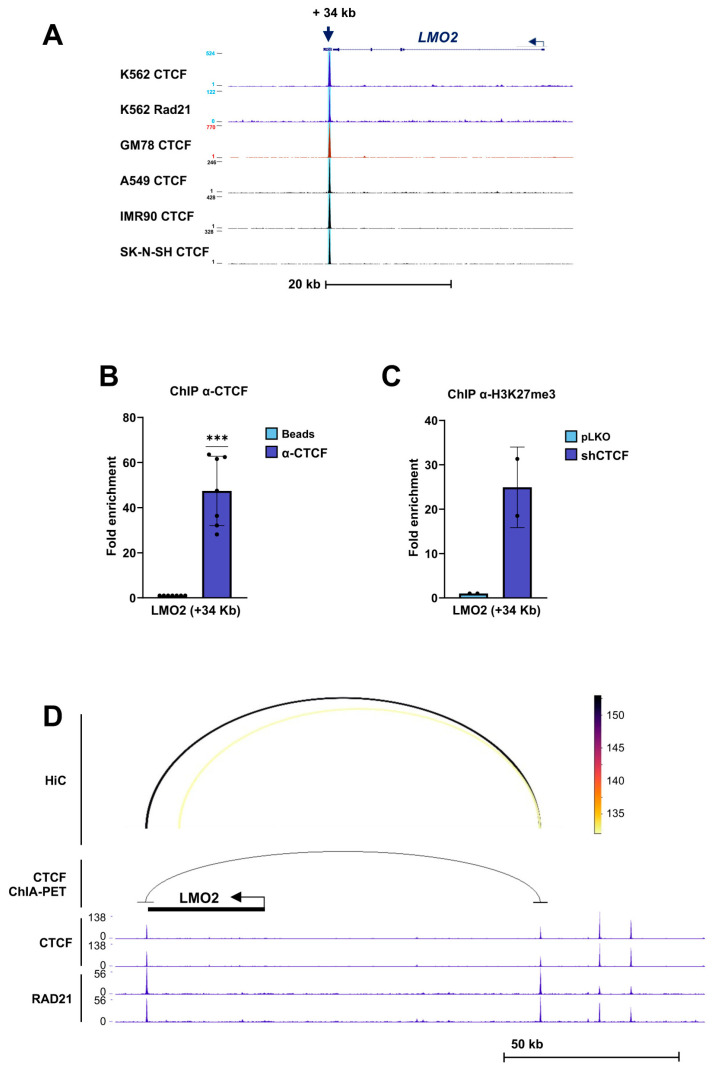
CTCF binding to the *LMO2* locus and the chromatin changes following CTCF downregulation. (**A**) ENCODE ChIP-seq profiles of CTCF and the cohesin subunit RAD21 across the *LMO2* locus on chromosome 11 in the indicated human cell lines. A conserved CTCF-binding site located approximately +34 kb downstream of the *LMO2* transcription start site is highlighted (blue arrow). (**B**) Chromatin immunoprecipitation (ChIP) analysis showing binding of CTCF to the *LMO2* CTCF-binding site (CTS) in K562 cells. Fold enrichment of the target region was calculated as described in the Section 2 and normalized to the no-antibody (beads-only) control. Data represent mean ± SD (*n* = 7). *** *p* < 0.001 by two-tailed one-sample *t*-test. (**C**) ChIP analysis of histone H3K27me3 enrichment at the *LMO2* CTS in K562 cells following infection with control lentivirus (pLKO, EV) or shRNA targeting CTCF (shCTCF). Data represent mean ± SD (*n* = 2). (**D**) Long-range chromatin interactions of the *LMO2* CTS (+34 kb), as identified by ENCODE CTCF ChIA-PET data and supported by Hi-C interaction profiles in K562 cells. ChIP-seq tracks for CTCF and RAD21 are shown. The interaction arc indicates a putative CTCF-mediated chromatin loop connecting the downstream CTS with an upstream CTCF-binding site at the *LMO2* locus.

For the *LMO2* locus, ChIP-seq analysis identified a prominent CTCF-binding site located approximately 34 kb downstream of the gene in K562 cells, which was conserved across all analyzed cell lines (Figure 5A). This site was also occupied by RAD21, indicating cohesin recruitment. ChIP assays confirmed robust CTCF binding at this site in K562 cells (Figure 5B). Upon CTCF silencing, ChIP analysis revealed a strong enrichment of H3K27me3 at the CTS, consistent with the acquisition of a repressive chromatin state (Figure 5C).

To explore whether this effect could involve recruitment of Polycomb repressive complex 2 (PRC2), we analyzed ENCODE ChIP-seq datasets for the core PRC2 components EZH2 and SUZ12 in K562 cells. Only modest overlap between PRC2 components and CTCF binding sites was observed genome-wide and at transcription start sites (Supplementary Figure S6), suggesting that PRC2 co-occupancy is restricted to a subset of loci.

To determine whether the increase in H3K27me3 observed reflects a global or locus-specific effect, we assessed global H3K27me3 levels by Western blot following CTCF depletion. No global increase in H3K27me3 was detected (Supplementary Figure S7), indicating that the accumulation of this repressive mark is restricted to specific genomic regions rather than representing a widespread chromatin change.

Analysis of ENCODE CTCF ChIA-PET data, together with Hi-C interaction profiles in K562 cells, revealed long-range chromatin interactions between this downstream CTS and a second CTCF-binding site located upstream of the *LMO2* gene (Figure 5D). The convergence of ChIA-PET and Hi-C data suggests the formation of a CTCF-mediated chromatin loop linking distal regulatory regions to the *LMO2* locus, potentially contributing to the regulation of *LMO2* expression.

**Figure 6:**
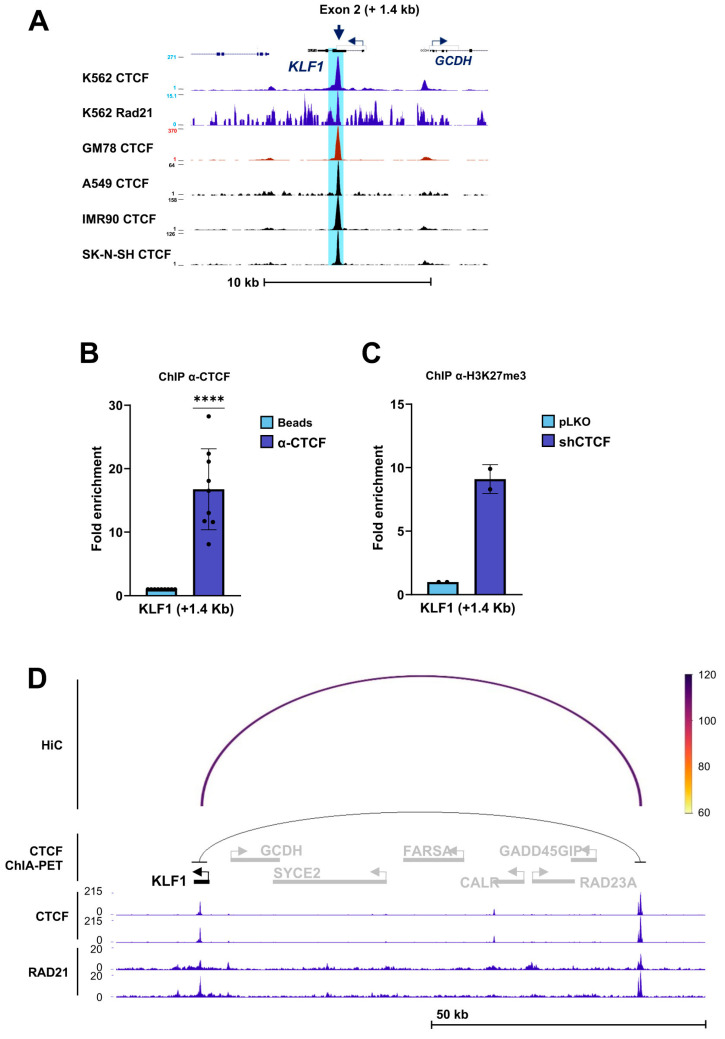
CTCF binding to *KLF1* and the chromatin changes following CTCF downregulation. (**A**) ENCODE ChIP-seq profiles of CTCF and the cohesin subunit RAD21 across the *KLF1* locus on chromosome 19 in the indicated human cell lines. A conserved CTCF-binding site located approximately +1.4 kb relative to the *KLF1* transcription start site is highlighted (blue arrow). (**B**) Chromatin immunoprecipitation (ChIP) analysis showing binding of CTCF to the *KLF1* CTCF-binding site (CTS) in K562 cells. Fold enrichment of the target region was calculated as described in the Section 2 and normalized to the no-antibody (beads-only) control. Data represent mean ± SD (*n* = 9). **** *p* < 0.0001 by two-tailed one-sample *t*-test. (**C**) ChIP analysis of histone H3K27me3 enrichment at the *KLF1* CTS in K562 cells following infection with control lentivirus (pLKO, EV) or shRNA targeting CTCF (shCTCF). Data represent mean ± SD (*n* = 2). (**D**) Long-range chromatin interactions of the *KLF1* CTS (+1.4 kb), as identified by ENCODE CTCF ChIA-PET data and supported by Hi-C interaction profiles in K562 cells. ChIP-seq tracks for CTCF and RAD21 are shown. The interaction arc indicates a putative CTCF-mediated chromatin loop linking the exon 2 CTS with an upstream CTCF-binding site.

Similarly, ENCODE ChIP-seq analysis identified a CTCF/RAD21-binding site located within exon 2 of the *KLF1* gene in all analyzed cell lines (Figure 6A). ChIP assays confirmed robust CTCF occupancy at this site (Figure 6B). Upon CTCF knockdown, a marked increase in H3K27me3 enrichment was detected at the exon 2 CTS, indicating the acquisition of a repressive chromatin state (Figure 6C). Analysis of ENCODE CTCF ChIA-PET data, together with Hi-C interaction profiles in K562 cells, revealed a long-range chromatin interaction between this exon 2 CTS and a second CTS located upstream of the *KLF1* locus (Figure 6D). This interaction provides support for the formation of a CTCF-mediated chromatin loop that may regulate *KLF1* expression.

**Figure 7:**
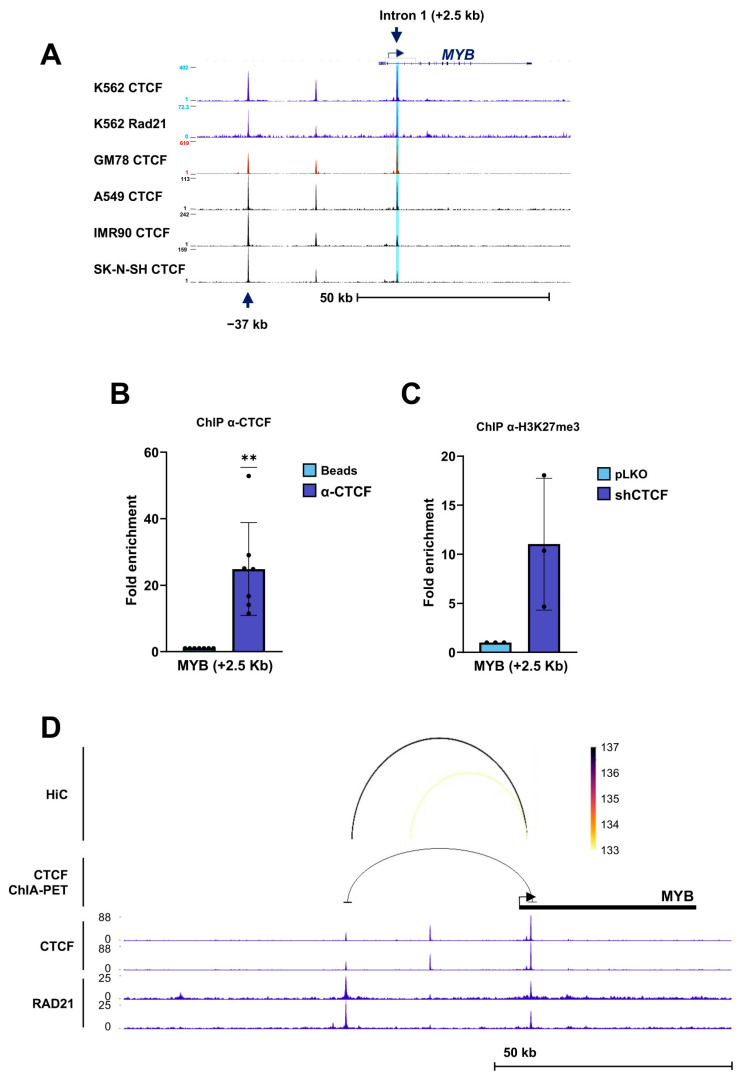
CTCF binding to *MYB* and the chromatin changes following CTCF downregulation. (**A**) ENCODE ChIP-seq profiles of CTCF and the cohesin subunit RAD21 across the *MYB* locus on chromosome 6 in the indicated human cell lines. A conserved CTCF-binding site located approximately +2.5 kb relative to the *MYB* transcription start site is highlighted (blue arrow). (**B**) Chromatin immunoprecipitation (ChIP) analysis showing binding of CTCF to the *MYB* CTCF-binding site (CTS) in K562 cells. Fold enrichment of the target region was calculated as described in the Section 2 and normalized to the no-antibody (beads-only) control. Data represent mean ± SD (*n* = 7). ** *p* < 0.01 by two-tailed one-sample *t*-test. (**C**) ChIP analysis of histone H3K27me3 enrichment at the *MYB* CTS in K562 cells following infection with control lentivirus (pLKO, EV) or shRNA targeting CTCF (shCTCF). Data represent mean ± SD (*n* = 3). (**D**) Long-range chromatin interactions involving the *MYB* CTS (+2.5 kb), as identified by ENCODE CTCF ChIA-PET data and supported by Hi-C interaction profiles in K562 cells. ChIP-seq tracks for CTCF and RAD21 are shown. The interaction arc indicates a putative CTCF-mediated chromatin loop connecting the intronic CTS with an upstream CTCF-binding site.

At the *MYB* locus, ENCODE ChIP-seq data identified a conserved CTCF/RAD21-binding site within intron 1 of the gene (Figure 7A). ChIP assays confirmed strong CTCF occupancy at this site (Figure 7B). Upon CTCF depletion, ChIP analysis revealed an enrichment of the repressive histone mark H3K27me3 at the intron 1 CTS, consistent with transcriptional repression (Figure 7C). Analysis of ENCODE CTCF ChIA-PET data, together with Hi-C interaction profiles in K562 cells, identified a long-range chromatin interaction between this intronic CTS and a second CTCF-binding site located approximately 37 kb upstream of the *MYB* transcription start site (Figure 7D). The convergence of ChIA-PET and Hi-C signals suggests the presence of a CTCF-mediated chromatin loop that may contribute to the regulation of *MYB* expression.

**Figure 8:**
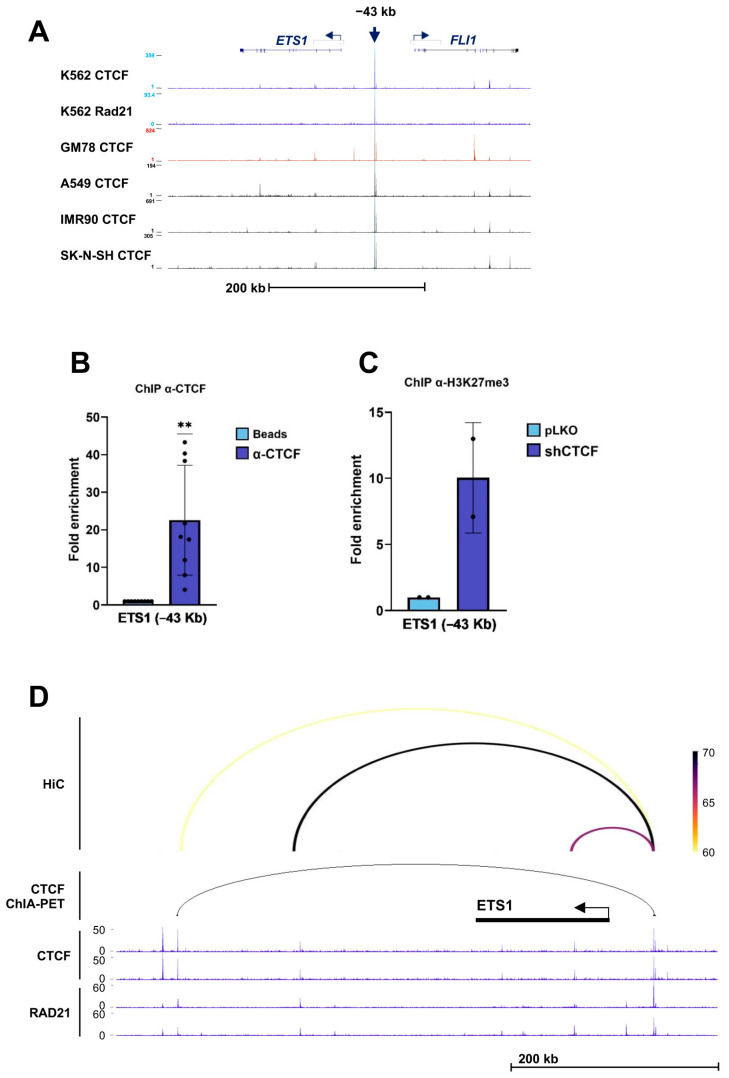
CTCF binding to *ETS1* and the chromatin changes following CTCF downregulation. (**A**) ENCODE ChIP-seq profiles of CTCF and the cohesin subunit RAD21 across the *ETS1* locus on chromosome 11 in the indicated human cell lines. A conserved CTCF-binding site located approximately −43 kb relative to the *ETS1* transcription start site is highlighted (blue arrow). (**B**) Chromatin immunoprecipitation (ChIP) analysis showing binding of CTCF to the *ETS1* CTCF-binding site (CTS) in K562 cells. Fold enrichment of the target region was calculated as described in the Section 2 and normalized to the no-antibody (beads-only) control. Data represent mean ± SD (*n* = 9). ** *p* < 0.01 by two-tailed one-sample *t*-test. (**C**) ChIP analysis of histone H3K27me3 enrichment at the *ETS1* CTS in K562 cells following infection with control lentivirus (pLKO, EV) or shRNA targeting CTCF (shCTCF). Data represent mean ± SD (*n* = 2). (**D**) Long-range chromatin interactions involving the *ETS1* CTS (−43 kb), as identified by ENCODE CTCF ChIA-PET data and supported by Hi-C interaction profiles in K562 cells. ChIP-seq tracks for CTCF and RAD21 are shown. The interaction arcs indicate putative CTCF-mediated chromatin loops linking upstream and downstream CTSs, consistent with an insulating regulatory configuration between *ETS1* and the neighboring *FLI1* locus.

Finally, at the *ETS1* locus, ENCODE ChIP-seq analysis identified a conserved CTCF/RAD21-binding site located approximately 43 kb upstream of the gene in K562 cells (Figure 8A). ChIP assays confirmed CTCF occupancy at this site in vivo (Figure 8B), while CTCF knockdown resulted in increased H3K27me3 enrichment, indicating the establishment of a repressive chromatin environment (Figure 8C). Analysis of ENCODE CTCF ChIA-PET data, together with Hi-C interaction profiles, revealed a chromatin interaction between this upstream CTS and a downstream CTS within the locus (Figure 8D). Notably, the inferred looping configuration suggests that CTCF may function as an insulating element separating *ETS1* from the neighboring ETS-family gene *FLI1*, thereby contributing to locus-specific transcriptional regulation.

Taken together, these data show that CTCF directly binds regulatory regions of key erythroid transcription factor genes and contributes to the maintenance of an active chromatin environment by preventing the accumulation of repressive histone marks and by organizing long-range chromatin interactions.

## 4. Discussion

Erythropoiesis is a highly dynamic and tightly regulated multistep process that drives the differentiation of hematopoietic stem cells into mature red blood cells. In our previous studies, we demonstrated that CTCF expression is modulated during differentiation across different hematopoietic lineages, including erythroid, megakaryocytic, granulocytic and monocytic cells [25]. We also showed that CTCF overexpression specifically promotes erythroid differentiation in pluripotent cells without affecting other lineages [26]. Together, these findings provided the first evidence for CTCF in regulating hematopoietic cell fate decisions. In the present study, we investigated the role of CTCF in erythroid differentiation using two complementary and widely used models [1]: pluripotent K562 leukemia cells and primary human CD34^+^ hematopoietic stem/progenitor cells, both induced to undergo erythroid differentiation by distinct stimuli. Our findings reveal that: (i) in both constitutive and inducible models, CTCF downregulation significantly impairs erythroid differentiation; (ii) CTCF silencing leads to repression of key erythroid transcription factor genes, including *LMO2*, *KLF1*, *MYB* and *ETS1*, through enrichment of the repressive histone mark H3K27me3 at CTCF binding sites within their regulatory regions; (iii) gene expression profiling in cells overexpressing CTCF identified enrichment of genes involved in erythroid lineage, consistent with the induction of erythroid differentiation [26]; and (iv) CTCF, in cooperation with cohesin, contributes to the establishment of long-range chromatin interactions at these erythroid gene loci. Importantly, these effects are not associated with a global loss of cell viability or widespread chromatin repression, supporting a locus-specific regulatory role of CTCF in erythroid gene expression.

### 4.1. CTCF Downregulation Impairs Erythroid Cell Differentiation

CTCF expression was reduced by lentiviral transduction with shCTCF using two complementary strategies: a constitutive knockdown vector and a doxycycline-inducible system. Across conditions, both approaches achieved a ~60–80% decrease in CTCF levels. We first evaluated the functional consequences of CTCF depletion in K562 cells by transducing cells and subsequently treating them with Ara-C or imatinib to promote erythroid differentiation. CTCF downregulation led to a marked reduction in the proportion of hemoglobin-producing cells and was accompanied by decreased expression of key erythroid markers, including γ-globin and GATA 1, indicating impaired erythroid differentiation. These results are consistent with prior work reporting reduced γ-globin transcription following CTCF depletion [38]; however, in contrast to our findings, that study did not detect changes in GATA 1 expression. Because those data were generated in an erythroid cell line, we next asked whether the phenotype could be reproduced in primary human CD34^+^ hematopoietic cells. Using both lentiviral systems, we achieved efficient CTCF knockdown in primary human CD34^+^ hematopoietic stem/progenitor cells and examined erythropoietin induced erythroid differentiation. CTCF depletion significantly reduced the proportion of hemoglobin-producing cells, decreased the number of glycophorin A-positive cells, and lowered γ-globin and GATA1 protein levels, further supporting a role of CTCF in promoting erythropoiesis. Collectively, our data show that CTCF downregulation impairs both spontaneous and induced erythroid differentiation in K562 cells and in CD34^+^ hematopoietic stem/progenitor cells. CTCF silencing has also been examined in other hematopoietic settings. Conditional deletion of CTCF has been used to probe its contribution to chromatin looping at the murine β-globin locus [39] and hematopoietic-specific CTCF knockout in mice has been associated with severe bone marrow failure and hematopoietic stem cell depletion [27]. Importantly, the consequences of CTCF loss appear to be lineage-dependent. For example, CTCF depletion in murine bone marrow enhances differentiation of common myeloid progenitors, suggesting a distinct—and potentially repressive—role for CTCF in macrophage lineage commitment [40]. Recently, Yang et al. reported the effects of acute CTCF loss in erythroid cells using an auxin-inducible degron system in different cell models [31]. Their data demonstrated that CTCF is required for erythroid maturation, in agreement with our findings. However, while Yang et al. focused on genome-wide chromatin accessibility, our study specifically links CTCF to the regulation of erythroid transcription factor programs.

Our results indicate that the impaired erythroid differentiation observed upon CTCF depletion is not a secondary consequence of reduced cell viability or increased apoptosis. Viability assays performed in both K562 and CD34^+^ cells showed no major loss of cell viability, and analysis of PARP1 cleavage did not reveal increased apoptosis upon CTCF knockdown. These findings argue against a general collapse of cellular activity and support a specific role for CTCF in regulating erythroid differentiation programs. Furthermore, flow cytometry analyses of CD34^+^ cells revealed that CTCF-depleted cells retain higher cellular complexity and size within the glycophorin A-positive population, consistent with an immature phenotype and impaired erythroid maturation.

### 4.2. CTCF Directly Regulates Erythroid Transcription Factor Genes

Erythropoiesis is a tightly regulated developmental process controlled by coordinated transcription factor activity, epigenetic modifications, and higher-order chromatin organization [4,41]. CTCF has emerged as a multifunctional regulator integrating these layers of gene regulation. Genomic studies have shown that CTCF and cohesin co-occupy regulatory regions in hematopoietic stem and erythroid cells, where they are associated with dynamic changes in chromatin architecture and transcriptional output during differentiation [42]. In this study, we define a direct and gene-specific role for CTCF in erythropoiesis by demonstrating that CTCF regulates the expression of key erythroid transcription factors through maintenance of permissive chromatin states and organization of long-range chromatin interactions.

Our global gene expression profiling revealed that modulation of CTCF levels profoundly affects erythroid transcriptional programs (Figure 3). Notably, both gain- and loss-of-function approaches indicate that erythroid differentiation is highly sensitive to CTCF dosage. Importantly, multiple lines of evidence argue against a global collapse of transcription upon CTCF depletion. Our transcriptomic analysis reveals both upregulated and downregulated gene sets, consistent with a selective regulatory role of CTCF. In agreement with previous studies showing that acute CTCF depletion leads to both upregulation and downregulation of specific gene sets [43,44,45], we did not observe a global increase in H3K27me3 levels upon CTCF knockdown. The absence of global changes in this repressive histone mark further supports that CTCF depletion does not promote a widespread transcriptional shutdown, but rather results in locus-specific alterations in gene expression.

Building on these findings, we focused on four key erythroid transcriptional regulators—*LMO2*, *KLF1*, *MYB*, and *ETS1*—and systematically examined whether CTCF directly regulates their expression through binding to defined regulatory regions. Using ENCODE datasets, we identified conserved CTCF-binding sites associated with each locus and validated CTCF occupancy by ChIP. Importantly, CTCF silencing consistently led to enrichment of the repressive histone mark H3K27me3 at these sites, indicating that CTCF is required to maintain an active chromatin environment. Notably, this effect appears to be locus-specific, as assessment of global H3K27me3 levels by Western blot did not reveal an overall increase upon CTCF knockdown. This finding indicates that the accumulation of H3K27me3 at erythroid gene loci reflects localized chromatin remodeling rather than a widespread epigenetic change. Integration of ChIA-PET and Hi-C datasets further revealed that these CTCF-bound regions participate in long-range chromatin interactions, supporting a direct role for CTCF in shaping the regulatory architecture of erythroid genes.

*LMO2* is a non-DNA-binding component of the core erythroid network and a member of the transcriptional “heptad” that orchestrates hematopoiesis [14]. Its expression increases during erythroid differentiation [46], a pattern we confirmed in our experimental models. We show that CTCF depletion markedly reduces *LMO2* expression both at baseline and during erythroid differentiation, consistent with previous findings showing that *LMO2* knockdown impairs erythroid differentiation by downregulating key erythroid genes [46]. We identified a conserved CTCF/RAD21-binding site located 34 kb downstream of the *LMO2* transcription start site and demonstrated that loss of CTCF leads to increased H3K27me3 enrichment at this site. ChIA-PET and Hi-C analyses indicate that this downstream CTS engages in long-range interactions with upstream CTCF-bound regions, suggesting the formation of a regulatory chromatin loop that supports *LMO2* transcription. This model is consistent with findings in murine erythroid cells, where multiple CTCF/cohesin-bound regions upstream of *Lmo2* participate in chromatin looping [47]. Notably, disruption of CTCF-mediated loop boundaries at the *LMO2* locus has been implicated in aberrant *LMO2* activation in T-cell acute lymphoblastic leukemia [48,49], underscoring the broader relevance of CTCF-dependent chromatin architecture in both normal erythropoiesis and malignant transformation.

*KLF1* is a master regulator of erythroid differentiation and β-globin gene expression [9,50]. It directly and indirectly regulates the majority of genes involved in the terminal stages of erythropoiesis [50]. In agreement with its established role [9], *KLF1* expression increased following erythroid induction in our K562 model, whereas CTCF depletion impaired its induction. We identified a conserved CTCF/RAD21-binding site within exon 2 of *KLF1* and demonstrated that CTCF loss results in increased H3K27me3 enrichment at this site. ChIA-PET and Hi-C analyses revealed a chromatin interaction between the exon 2 CTS and an upstream CTCF-bound region. These findings align with recent studies in murine erythroid cells showing that CTCF cooperates with DDX5 at exon 2 to form short-range chromatin loops essential for *Klf1* transcription [51]. Together, these data support a conserved mechanism whereby CTCF binding at exon 2 maintains an active chromatin configuration necessary for *KLF1* expression.

*MYB* is a central regulator of hematopoiesis whose expression must be tightly controlled, as sustained *MYB* activity impairs erythroid differentiation and aberrant *MYB* expression has been reported in various human leukemias and lymphomas [1,52]. Consistent with previous reports, we observed downregulation of *MYB* during erythroid differentiation [53]. We identified a conserved CTCF/RAD21-binding site within intron 1 of *MYB* and showed that CTCF depletion leads to increased H3K27me3 enrichment at this site. ChIA-PET and Hi-C data revealed interactions between this intronic CTS and an upstream regulatory region corresponding to the −36 kb enhancer previously described by Stadhouders et al. [53]. These findings support a model in which CTCF maintains *MYB* expression in undifferentiated cells by stabilizing enhancer–promoter communication, while loss of CTCF facilitates chromatin repression and transcriptional downregulation during erythroid differentiation.

Finally, *ETS1* encodes an ETS-family transcription factor with key roles in hematopoiesis, stem cell maintenance, and cancer [54]. *ETS1* contributes to erythroid and megakaryocytic lineage specification and is downregulated during erythroid differentiation [11], a pattern recapitulated in our model. We identified a conserved CTCF/RAD21-binding site located 43 kb upstream of the *ETS1* gene and demonstrated that CTCF depletion leads to increased H3K27me3 enrichment at this site and reduced *ETS1* expression. ChIA-PET and Hi-C analyses revealed interactions linking this upstream CTS to downstream CTCF-bound regions within the locus. Notably, this CTS is positioned between *ETS1* and *FLI1*, another ETS-family gene with critical roles in hematopoiesis and leukemogenesis [12]. The inferred interaction pattern suggests that CTCF may function as an insulating element that contributes to the coordinated and lineage-specific regulation of *ETS1* and *FLI1* during erythropoiesis.

Notably, the CTCF-binding sites identified at the *LMO2*, *KLF1*, *MYB*, and *ETS1* loci are conserved across multiple cell types, consistent with the role of CTCF as a broadly expressed architectural protein. Therefore, CTCF binding at these loci is not erythroid-specific per se. Rather, our findings support a model in which the functional outcome of CTCF binding is context-dependent, and in erythroid cells, CTCF contributes to lineage-specific gene regulation through maintenance of local chromatin states and long-range chromatin interactions.

## 5. Conclusions

CTCF is a multifunctional transcriptional regulator with a central role in genome organization and gene regulation, and its involvement in cell proliferation and differentiation has been widely documented. Its frequent misregulation in cancer, including hematological tumors, underscores the need to define the molecular mechanisms through which CTCF controls lineage-specific gene expression programs. Erythroid differentiation represents a paradigmatic example of a developmental process requiring precise coordination between transcriptional and epigenetic regulatory layers.

In this study, we demonstrate that CTCF is required for proper erythroid differentiation, using complementary cellular models and different CTCF silencing approaches. Transcriptomic analyses identified key erythroid regulators whose expression is sensitive to CTCF levels, and focused mechanistic studies revealed that CTCF directly binds regulatory regions of core erythroid transcription factor genes. Loss of CTCF resulted in the accumulation of repressive chromatin marks at these loci and disruption of regulatory chromatin interactions, which lead to impaired expression of genes essential for erythropoiesis. Our data further indicate that these effects are locus-specific rather than global. CTCF depletion did not result in a major loss of cell viability or increased apoptosis, and no global increase in H3K27me3 levels was observed, supporting a model in which CTCF regulates chromatin states at selected genomic regions.

Collectively, these findings establish CTCF as a critical regulator of erythroid differentiation, functioning to maintain a permissive chromatin landscapes and proper regulatory interactions at key erythroid transcription factor genes. Notably, CTCF mutations and other alterations have been reported in several hematologic malignancies, and targeting chromatin looping has been proposed as a potential therapeutic strategy in leukemia.

## Data Availability

The original contributions presented in the study are included in the article/Supplementary Materials, and further inquiries can be directed to the corresponding authors. Gene expression profiling data are available in the Gene Expression Omnibus database; accession number GSE319471.

## Supplementary Materials

The following supporting information can be downloaded at: https://www.mdpi.com/article/10.3390/biom16040549/s1, Table S1: Primers used for RT-qPCR analysis; Table S2: Primary antibodies used for Western Blot; Table S3: Secondary antibodies used for Western Blot; Table S4: Antibodies used for ChIP experiments; Table S5: Primers used for ChIP experiments; Figure S1. Ara-C and Imatinib induce erythroid differentiation in K562 cells; Figure S2. Effect of CTCF knockdown on cell viability during erythroid differentiation; Figure S3. EPO induces erythroid differentiation in CD34^+^; Figure S4. CTCF downregulation inhibits erythroid differentiation in CD34^+^ cells; Figure S5. Effect of CTCF downregulation on erythroid genes protein levels; Figure S6. Genome-wide distribution of CTCF, and SUZ12, and EZH2 ChIP-seq signals; Figure S7. Global levels of H3K27me3 following CTCF depletion; Figure S8. Original Western blot images.

## Abbreviations

The following abbreviations are used in this manuscript:

Ara-C	1 μM 1-β-D-arabinofuranosylcytosine
ChIA-PET	Chromatin Interaction Analysis by Paired-End Tag Sequencing
ChIP	Chromatin Immunoprecipitation
ChIP-seq	Chromatin Immunoprecipitation followed by Sequencing
CTS	CTCF-binding site
Doxy	Doxycycline
ENCODE	Encyclopedia of DNA Elements
EPO	Erythropoietin
GEO	Gene Expression Omnibus
GO	Gene Ontology
Hi-C	High-throughput chromosome conformation capture
MOI	Multiplicity of infection
PCR	Polymerase Chain Reaction
RT-qPCR	Reverse transcription followed by quantitative PCR
SD	Standard Deviation
shRNA	short hairpin RNA
