# Supplementary material for: CTCF Regulates Erythroid Differentiation Through Control of Core Erythroid Transcription Factors
Source: Biomolecules. 2026 Apr 8;16(4):549. doi: 10.3390/biom16040549 (PMC13113740; doi:10.3390/biom16040549)

Supplementary Materials

CTCF regulates erythroid differentiation through control of core erythroid transcription factors

Lorena García-Gaipo, Vanessa Junco, Lucía García-Gutiérrez, Verónica Torrano, Rosa Blanco, Alexandra Weisinger, Rujula Pradeep, Jose Luis Arroyo, Ana Batlle-López, Javier León, Manuel Rosa-Garrido, M. Dolores Delgado

Table S1. Primers used for RT-qPCR analysis

AMPLIFIED GENE	PRIMER SEQUENCES (5´ → 3´)	AMPLICON SIZE
**CTCF**	Fw: TTACACGTGTCCACGGCGTTC<br>Rv: GCTTGTATGTGTCCCTGCTGGCA	365 bp
**ETS1**	Fw: TCCAGACAGACACCTTGCAG<br>Rv: TGAGGCGATCACAACTATCG	153 bp
**GYPA**	Fw: GAGAAAGGGTACAACTTGCC<br>Rv: CATTGATCACTTGTCTCTGG	220 bp
**HBE1**	Fw: GCAAGAAGGTGCTGACTTCC<br>Rv: TGCCAAAGTGAGTAGCCAGA	168 bp
**KLF1**	Fw: CAGGTGTGATAGCCGAGACC<br>Rv: CCGTGTGTTTCCGGTAGTG	241 bp
**LMO2**	Fw: CTGAGCTGCGACCTCTGTG<br>Rv: CGCATTGTCATCTCATAGGC	164 bp
**MYB**	Fw: AGCAAGGTGCATGATCGTC<br>Rv: GGGGGTGGAAGTTAAAGAAGG	157 bp
**RPS14**	Fw: TATCACCGCCCTACACATCA<br>Rv: GGGGTGACATCCTCAATCC	135 bp

Table S2. Primary antibodies used for Western Blot

ANTIBODY	TYPE	ORIGIN	DILUTION
**ACTIN (C-4)**	Mouse monoclonal	Santa Cruz BT (sc-47778)	1:3000
**CTCF**	Mouse monoclonal	BD Bioscience (612149)	1:1000
**ETS1**	Rabbit polyclonal	Cell Signaling (D808A)	1:500
**GATA1 (C-20)**	Goat polyclonal	Santa Cruz BT (sc-1233)	1:1000
**H3K27me**	Rabbit polyclonal	Active Motif (39155)	1:2000
**KLF1**	Mouse monoclonal	Santa Cruz BT (sc-166238)	1:1000
**LMO2**	Mouse monoclonal	Santa Cruz BT (sc-65736)	1:1000
**MYB**	Rabbit polyclonal	Cell Signaling (D2R4Y)	1:1000
**PARP1 (H-250)**	Rabbit polyclonal	Santa Cruz BT (sc-7150)	1:1000
**TUBULIN**	Rabbit polyclonal	Laboratory of Nick Cowan, NY, USA	1:3000
**γ-GLOBIN (51-7)**	Mouse monoclonal	Santa Cruz BT (sc-21756)	1:1000

Table S3. Secondary antibodies used for Western Blot

ANTIBODY	TYPE	ORIGIN	DILUTION
Anti-Goat IRDye®680	Donkey polyclonal	LI-COR (926-68074)	1:10000
Anti-Goat IRDye®800	Donkey polyclonal	LI-COR (926-32214)	1:10000
Anti-Mouse IRDye®680	Donkey polyclonal	LI-COR (926-68072)	1:10000
Anti-Mouse IRDye®800	Donkey polyclonal	LI-COR (926-32212)	1:10000
Anti-Rabbit IRDye®680	Donkey polyclonal	LI-COR (926-68073)	1:10000
Anti-Rabbit IRDye®800	Donkey polyclonal	LI-COR (926-32213)	1:10000

Table S4. Antibodies used for ChIP experiments

ANTIBODY	IMMUNOGEN	TYPE	ORIGIN
**CTCF**	Human CTCF aa. 184-290	Mouse monoclonal	BD Bioscience (612149)
**CTCF**	Human CTCF aa. 659-675	Rabbit polyclonal	Millipore (07-729)
**CTCF**	Human CTCF aa. 650-700	Rabbit polyclonal	Abcam (ab10571)
**H3K27me3**	anti-trimethyl-histone H3 (Lys27)	Rabbit polyclonal	Millipore (07-449)

Table S5. Primers used for ChIP experiments

AMPLIFIED GENE	LOCATION	PRIMER SEQUENCES (5´ → 3´)	AMPLICON SIZE
**ETS1**	-43 kb upstream	Fw: GAGGTCCTTCCTCCTGGAAC<br>Rv: ATGCAGCTATTGGGTTTTGC	184 bp
**KLF1**	Exon 2 (+ 1.4 kb)	Fw: GGTGGGAGCTCTTGGTGTAG<br>Rv: CCCCTCCTTCCTGAGTTGTT	191 bp
**LMO2**	34 kb downstream	Fw: TTAAGGTGATGGCCAGAAGG<br>Rv: TTTTCCAAGACGGGTGTCTC	162 bp
**MYB**	Intron 1 (+ 2.5 kb)	Fw: TCCAAGCAAGCCCTTATTGT<br>Rv: ACAACCCAGGAACAAGCAAC	198 bp

Negative and positive controls

**H4**	rDNA repeats	Fw: CGACGACCCATTCGAACGTCT<br>Rv: CTCTCCGGAATCGAACCCTGA	103 bp
**H42.1**	rDNA repeats	Fw: GCTTCTCGACTCACGGTTTC<br>Rv: CCGAGAGCACGATCTCAAA	124 bp

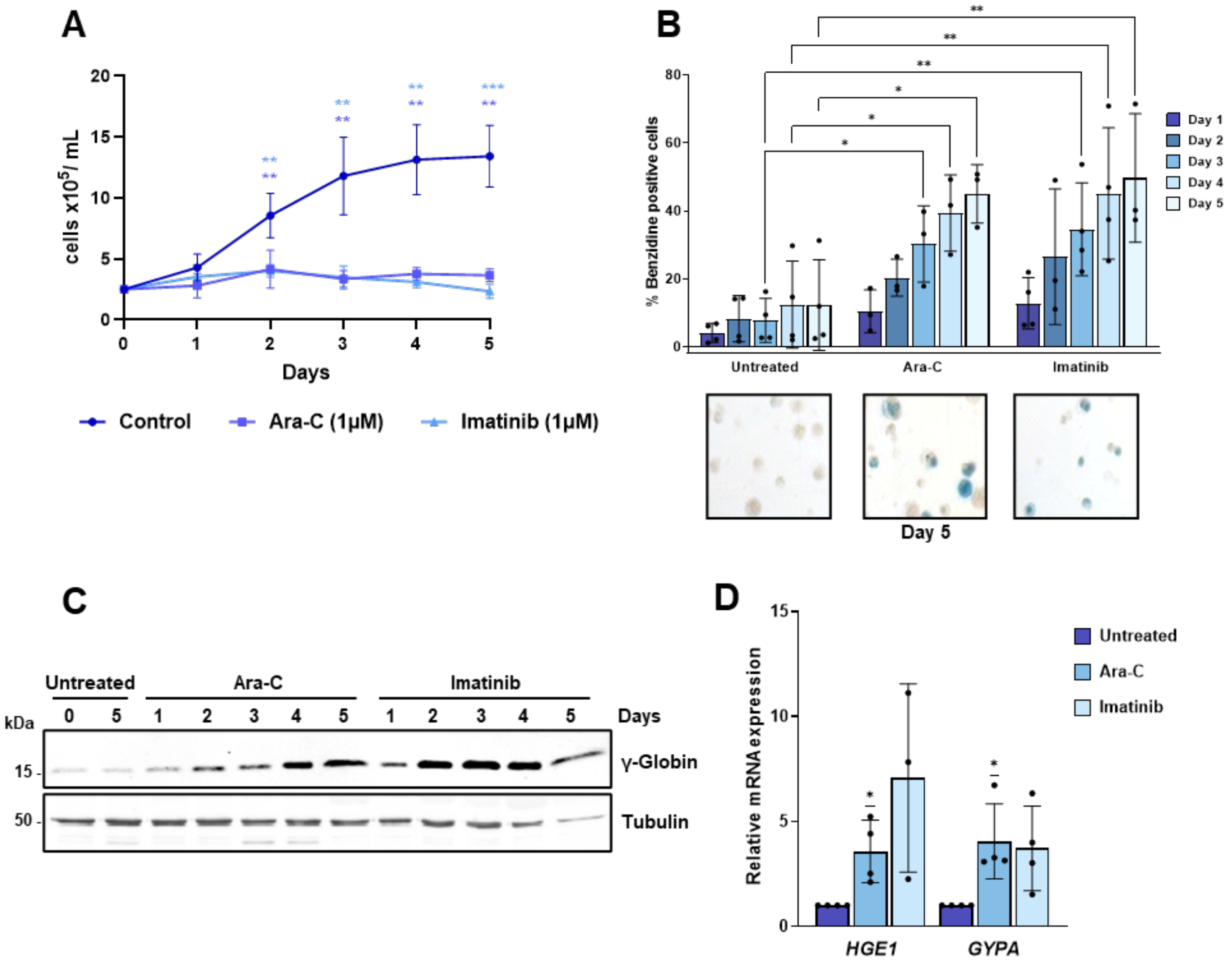

Figure S1. Ara-C and Imatinib induce erythroid differentiation in K562 cells. (**A**) Cell proliferation curves for K562. 2.5x10^5 cells were seeded and treated with 1 µM Ara-C or 1 µM Imatinib. Cells were counted daily for 5 days using a Nucleocounter. Data represent mean values ± SD (n = 5), ** *P*<0.01, *** *P*<0.001 by two-way ANOVA followed by Tukey's post hoc multiple comparisons test. (**B**) Benzidine test of K562 cells treated with 1 µM Ara-C or 1 µM imatinib for 5 days. Pictures taken during benzidine test evaluation are shown. In each experiment, a minimum of 200 cells were counted. Data represent mean values ± SD (n ≥ 3), * *P*<0.05, ** *P*<001 by two-way ANOVA followed by Dunnett's post hoc multiple comparisons test. (**C**) Protein expression of γ-globin analyzed by Western blot after treatment with 1 µM Ara-C and 1 µM imatinib for 5 days. Tubulin levels were used as loading control. (**D**) Expression of *HBE1* (ε-globin gene) and (*GYPA* (glycophorin-A gene) was analyzed by RT-qPCR after treatment with 1 µM Ara-C and 1 µM Imatinib for 72 hours. Expression was normalized against *RPS14* levels. Data represent mean values ± SD (n ≥ 3), * *P*<0.05 by one sample t-test (two tailed).

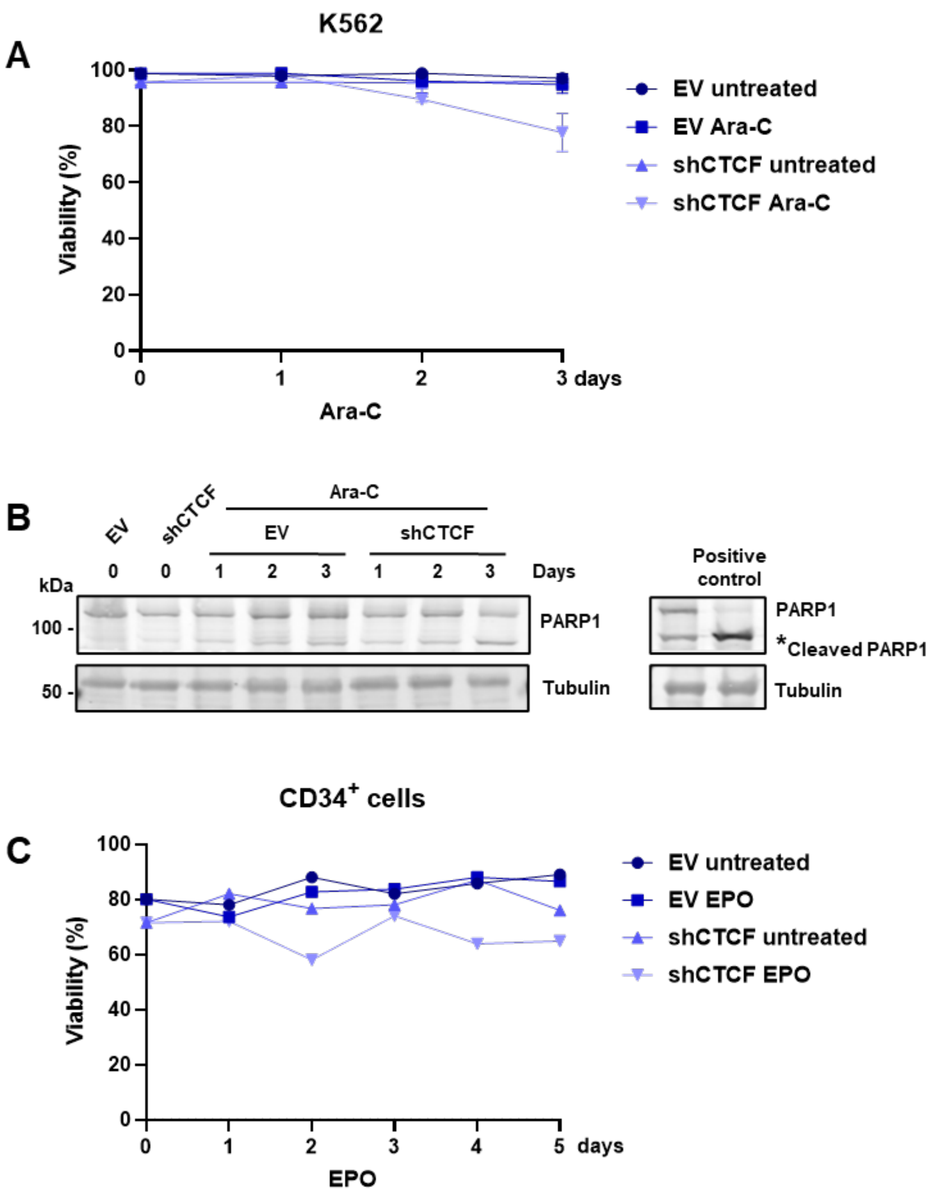

Figure S2. Effect of CTCF knockdown on cell viability during erythroid differentiation. (**A**) Viability of K562 cells following induction of erythroid differentiation with Ara-C. Cells transduced with pLKO empty vector (EV) or pLKO shCTCF were treated with Ara-C or left untreated, and viability was measured over 3 days using trypan blue exclusion. (**B**) Western blot analysis of PARP1 and cleaved PARP1 (asterisk, indicative of apoptosis) in K562 cells following infection with pLKO empty vector (EV) or pLKO shCTCF and treatment with 1 µM Ara-C for 3 days. Tubulin was used as a loading control. A positive control of cells undergoing apoptosis is shown. (**C**) Viability of human CD34^{+} hematopoietic progenitor cells during erythroid differentiation induced by erythropoietin (EPO). Cells transduced with pLKO empty vector (EV) or pLKO shCTCF were cultured in the presence or absence of EPO and viability was assessed daily for 5 days by trypan blue exclusion. Overall, CTCF knockdown does not result in a major loss of cell viability under these conditions.

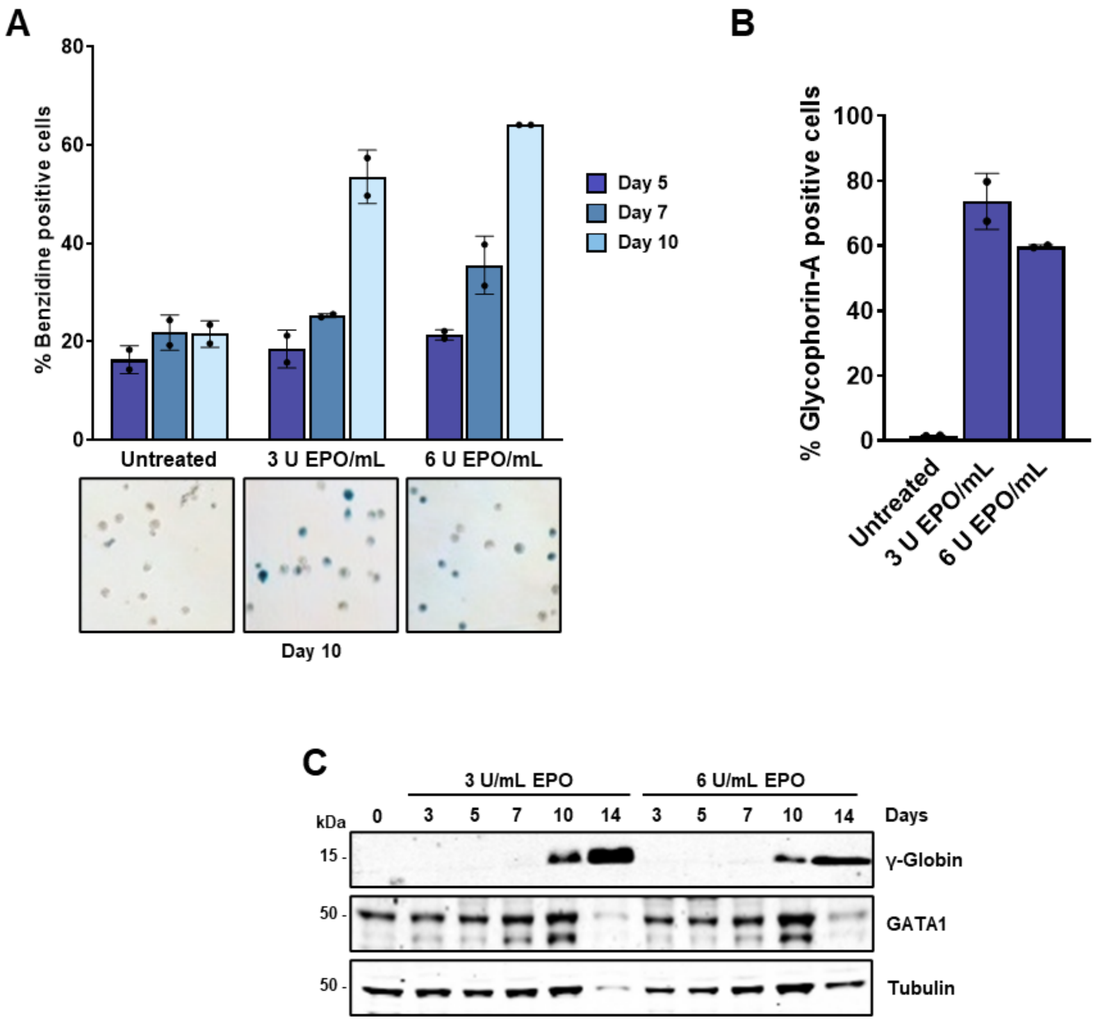

Figure S3. EPO induces erythroid differentiation in $CD34^+$. (**A**) Benzidine test of $CD34^+$ cells treated with 3 U/mL and 6 U/mL EPO during 10 days. Pictures taken during benzidine test evaluation are shown. In each experiment, a minimum of 200 cells were counted. Data represent mean values ± SD (n = 2). (**B**) Glycophorin A-positive cells analyzed by flow cytometry after treatment of $CD34^+$ cells with 3 U/mL and 6 U/mL EPO for 10 days. Data represent mean values ± SD (n = 2). (**C**) Protein expression of γ-globin and GATA1 analyzed by Western blot after treatment of $CD34^+$ cells with 3 U/mL and 6 U/mL EPO for 14 days. Tubulin levels were used as loading control.

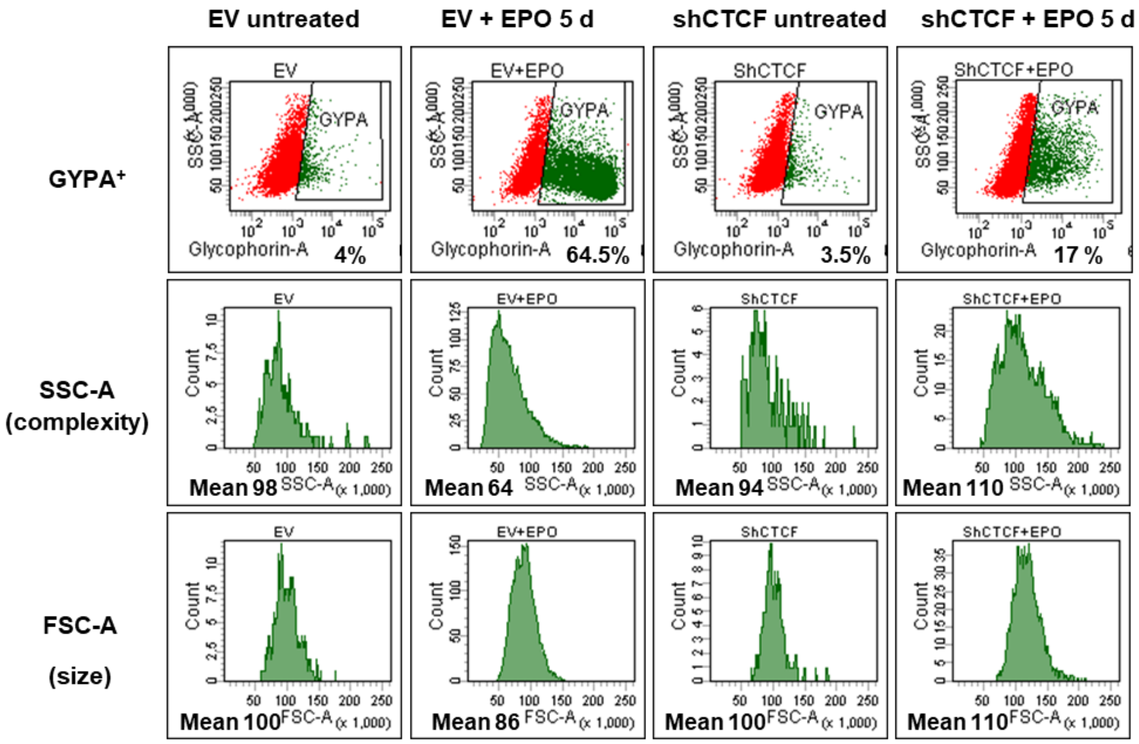

Figure S4. CTCF downregulation inhibits erythroid differentiation in CD34+ cells. Glycophorin A (GYPA)-positive cells were analyzed by flow cytometry following infection of CD34+ cells with pLKO empty vector (EV) or pLKO shCTCF and treatment with 3 U/mL erythropoietin (EPO) for 5 days. Representative flow cytometry plots showing GYPA-positive cells are presented in the upper panels. CTCF-depleted cells show a reduced proportion of GYPA-positive cells upon EPO treatment compared to control cells, indicating impaired erythroid differentiation. Middle and lower panels show the analysis of cellular complexity (SSC-A) and cell size (FSC-A), respectively, within the GYPA-positive population.

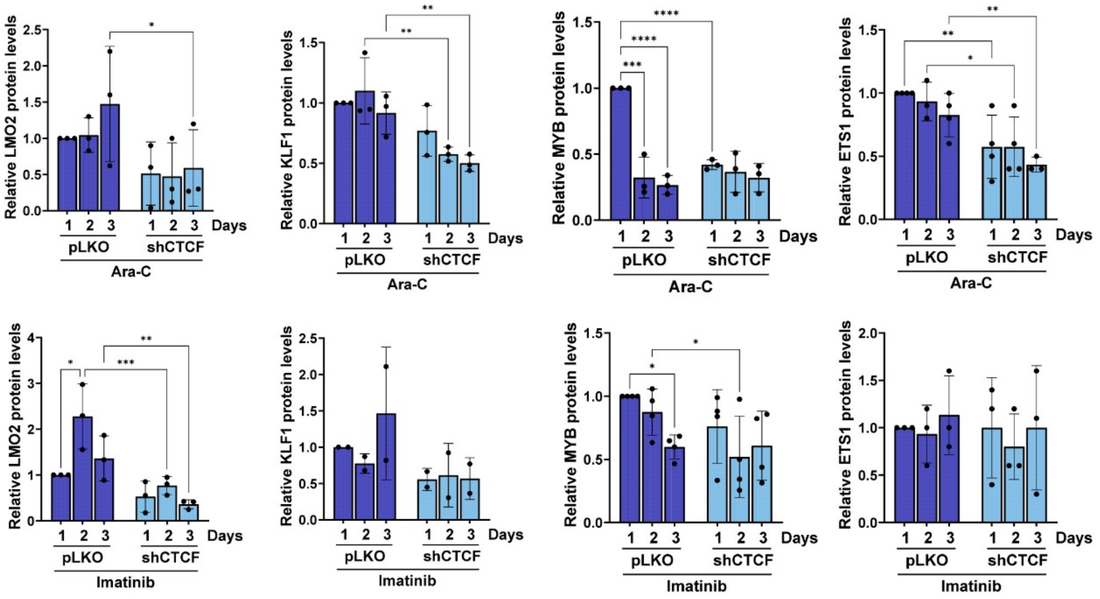

Figure S5. Effect of CTCF downregulation on erythroid genes protein levels**.** Protein expression of LMO2, KLF1, MYB and ETS1 analyzed by Western blot after infection of K562 cells with pLKO (EV) or pLKO shCTCF and treated with 1 µM Ara-C or 0.5 µM imatinib for 3 days. Protein signal quantification was normalized to the loading control. Data represent mean values ± SD (n≥3 except for KLF1 Imatinib n=2) *P > 0.05; ** P > 0.01; *** P > 0.001; **** P > 0.0001 by two-way ANOVA followed by Tukey's post hoc multiple comparisons test.

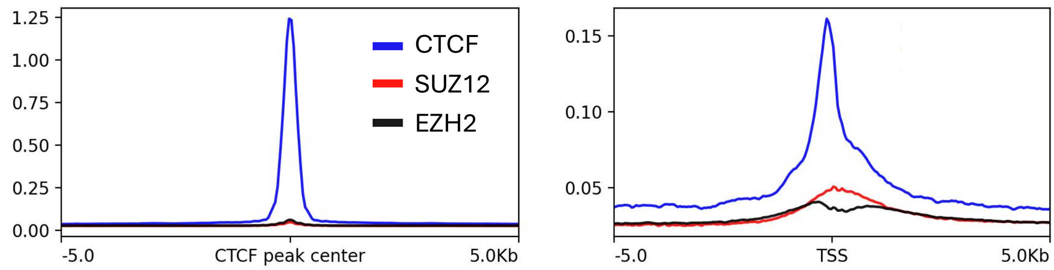

Figure S6. Genome-wide distribution of CTCF, and SUZ12, and EZH2 ChIP-seq signals. Metagene profiles showing the average ChIP-seq signal for CTCF, SUZ12, and EZH2 in K562 cells centered at CTCF peak regions (left panel) and transcription start sites (TSS; right panel). The x-axis represents genomic distance (kilobases, kb) relative to the center of CTCF peaks or TSS (±5 kb), and the y-axis represents normalized ChIP-seq signal intensity. CTCF exhibits a sharp and strong enrichment in both analyses, whereas SUZ12 and EZH2 display lower and broader signal distributions. The limited overlap between CTCF and PRC2 components at the genome-wide level suggests that any potential interaction between these factors is not global but may occur at specific genomic loci.

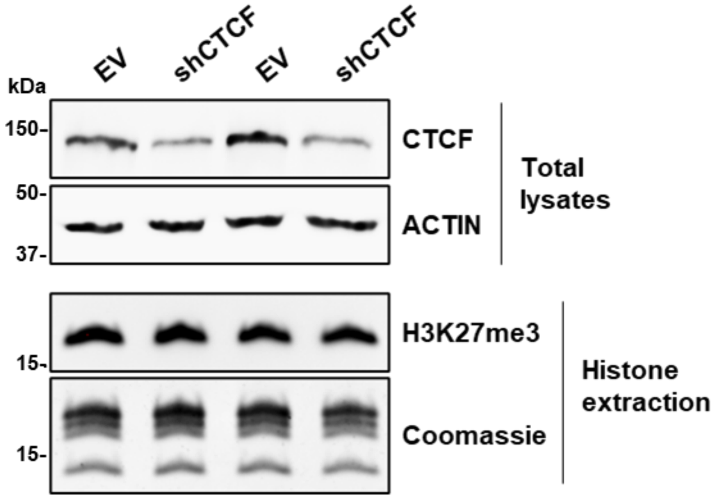

Figure S7. Global levels of H3K27me3 following CTCF depletion. Western blot in K562 cells transduced with the doxycycline-inducible pTRIPZ shCTCF construct or its corresponding empty vector (EV) following 2 µg/ml of Doxycyclin treatment for 3 days. Total cell lysates were used to assess CTCF protein levels, while histone-enriched extracts were prepared to evaluate H3K27me3 levels. Representative blots for CTCF, H3K27me3, and loading controls are shown. Despite efficient CTCF depletion, no global changes in H3K27me3 levels were observed, indicating that CTCF loss does not affect global H3K27 trimethylation.

Figure S8. Original Western Blot images. Full-length blots corresponding to the cropped images shown in Figures 1, 2, 4 and supplementary Figures S1, S2, S3 and S7. Molecular weight markers (M) and positive controls (C) are indicated in each Figure.

Original Western Blot images corresponding to Figure 1.

Figure 1 A

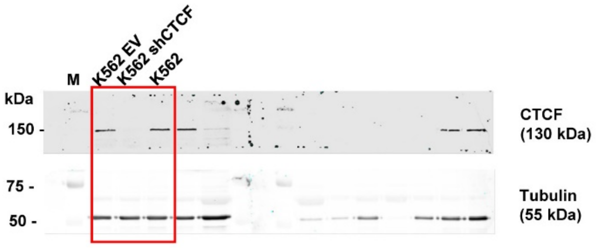

Figure 1 C

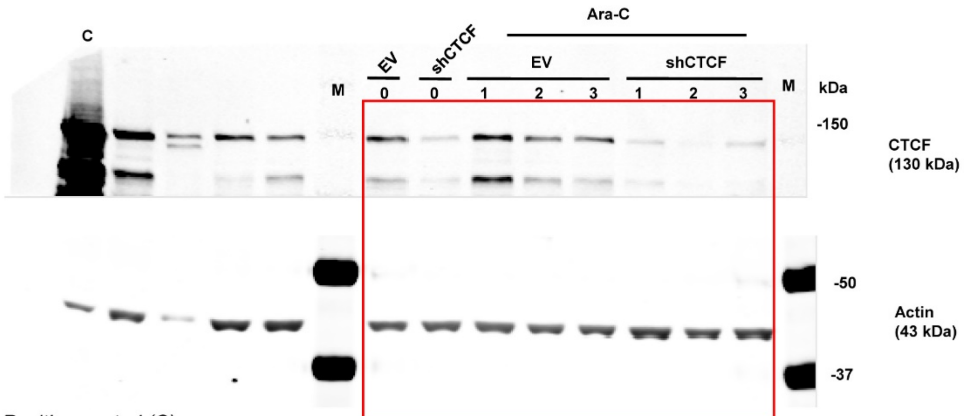

Positive control (C):
293T cells transfected
with pCDNA-CTCF

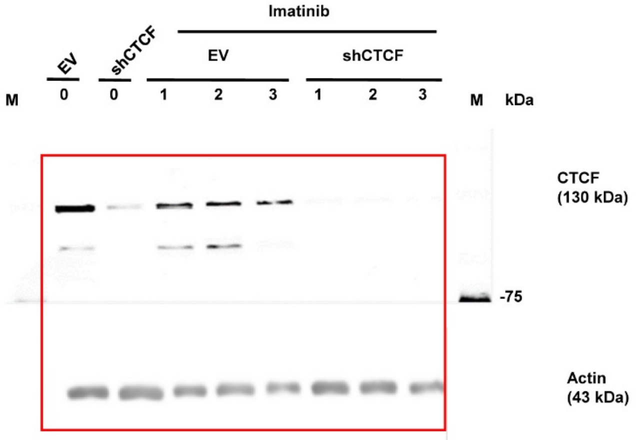

Original Western Blot images corresponding to Figure 1.

Figure 1 C

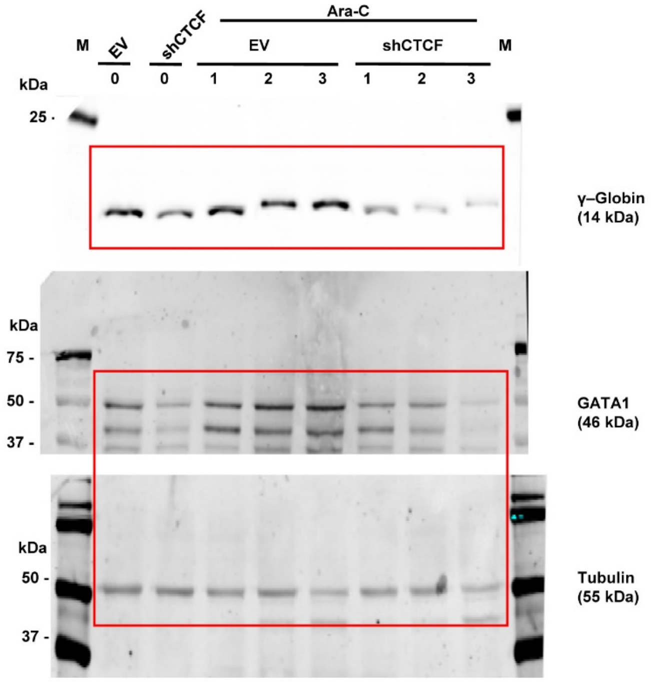

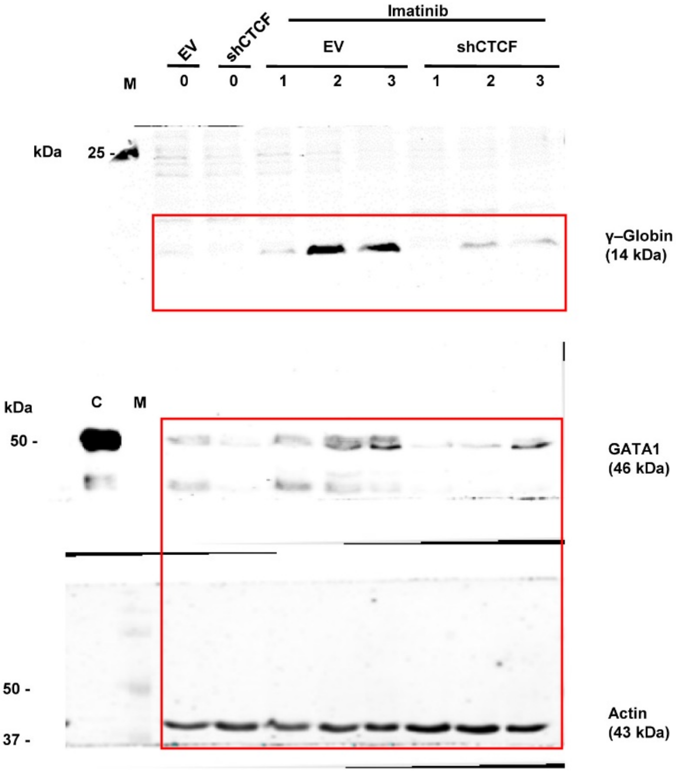

Positive control (C): 293T cells transfected with pCEFL-GATA1

Original Western Blot images corresponding to Figure 1.

Figure 1 F

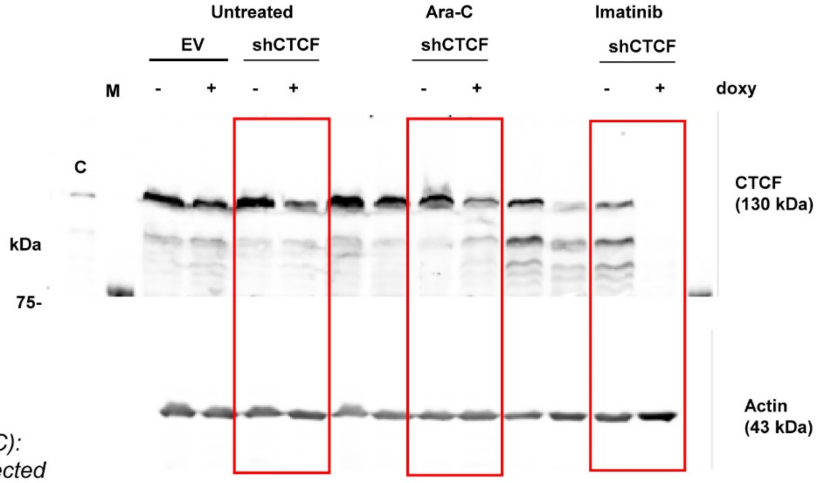

Positive control (C): 293T cells transfected with pCDNA-CTCF

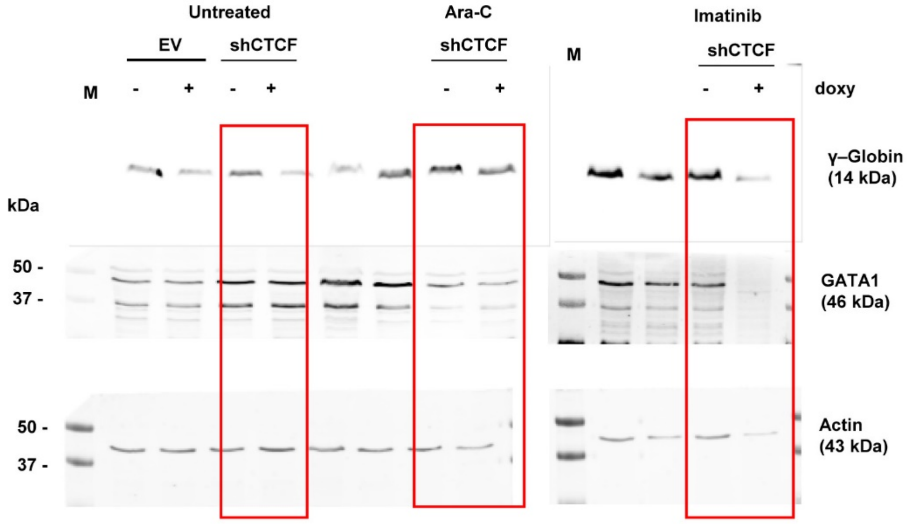

Original Western Blot images corresponding to Figure 2.

Figure 2 D

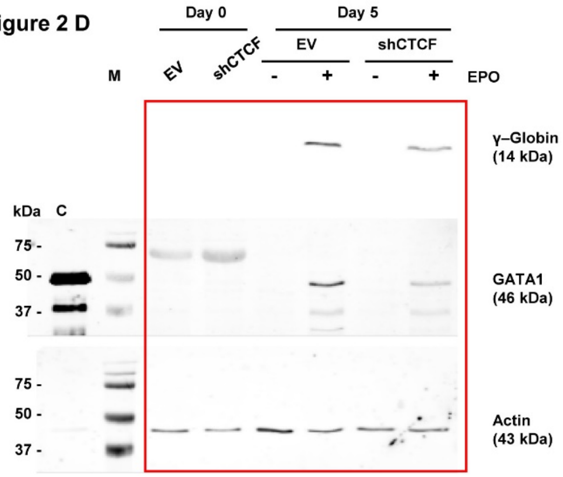

Positive control (C): 293T cells transfected with pCEFL-GATA1

Figure 2 G

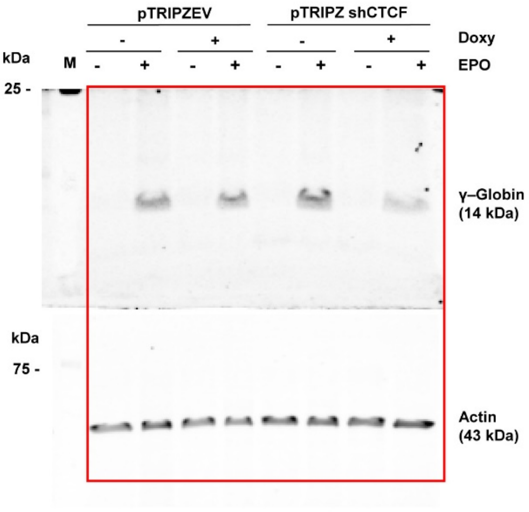

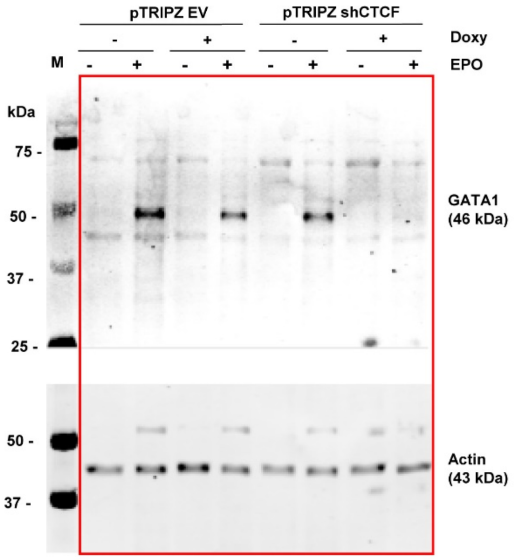

Original Western Blot images corresponding to Figure 4.

Figure 4D

LMO2 positive control (C): total lysate from Ramos cells.

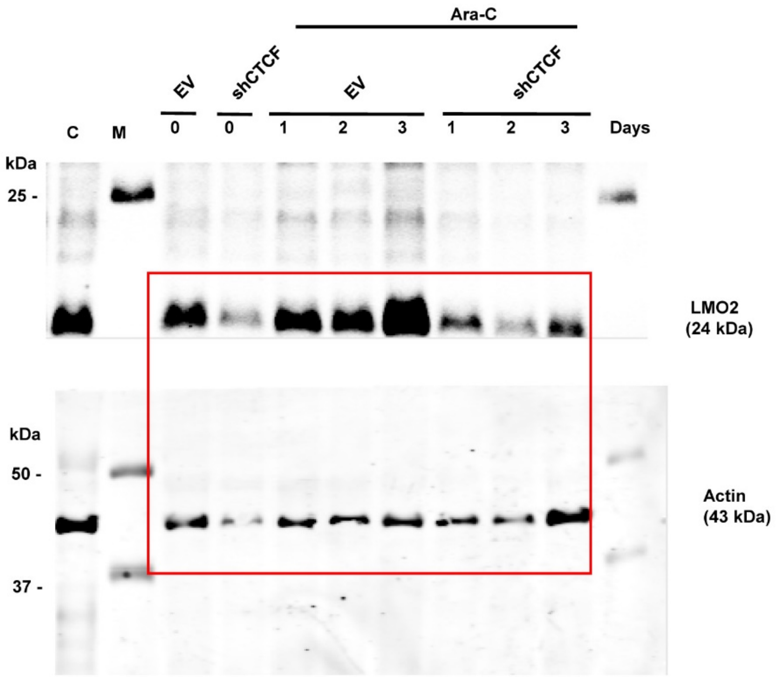

Figure 4E

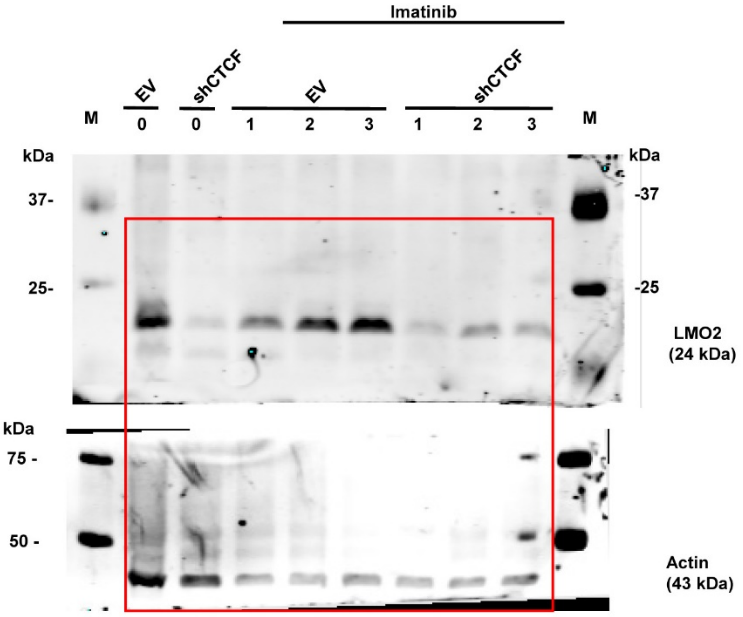

Original Western Blot images corresponding to Figure 4.

Figure 4D

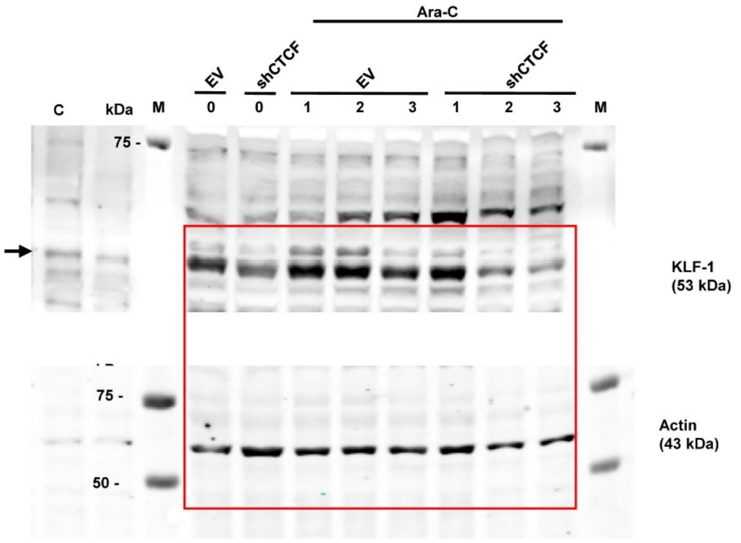

Figure 4E

KLF1 positive control (C):
total lysate from HeLa cells.

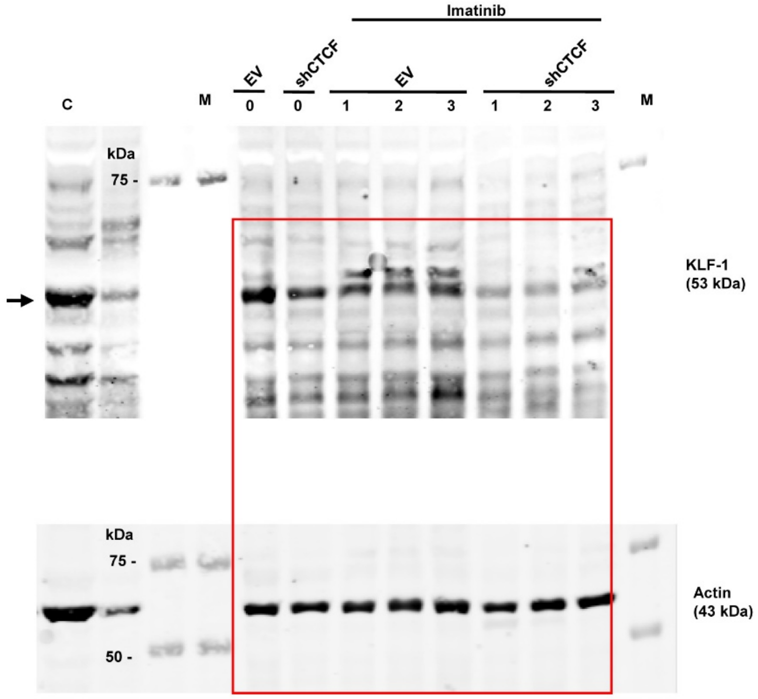

Original Western Blot images corresponding to Figure 4.

Figure 4D

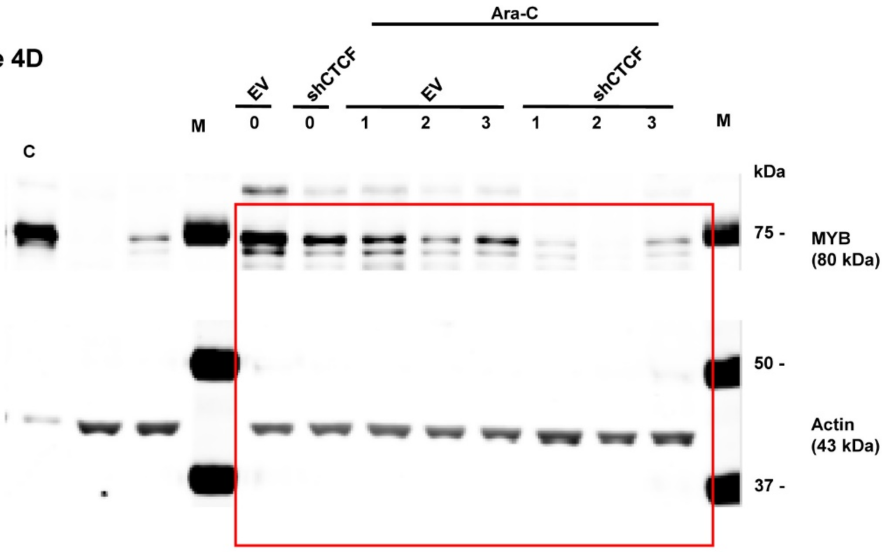

MYB positive control (C): total lysate from Jurkat cells.

Figure 4E

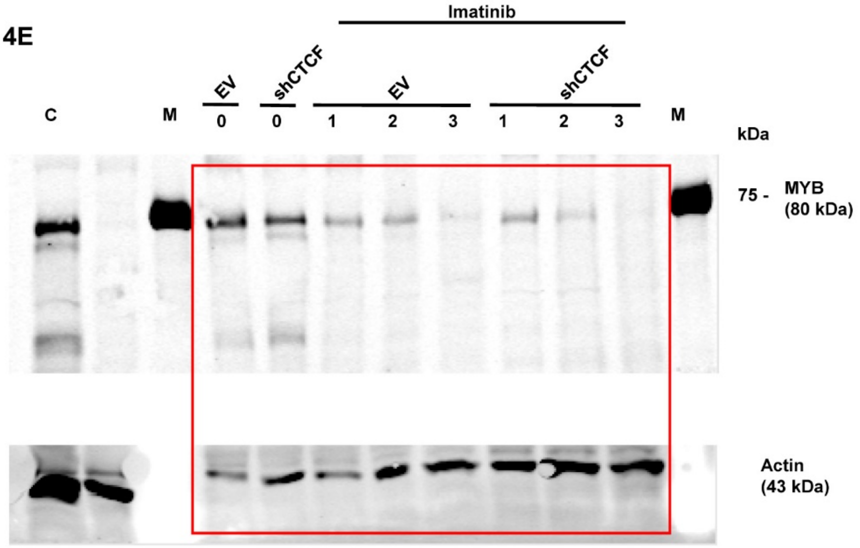

Original Western Blot images corresponding to Figure 4.

Figure 4D

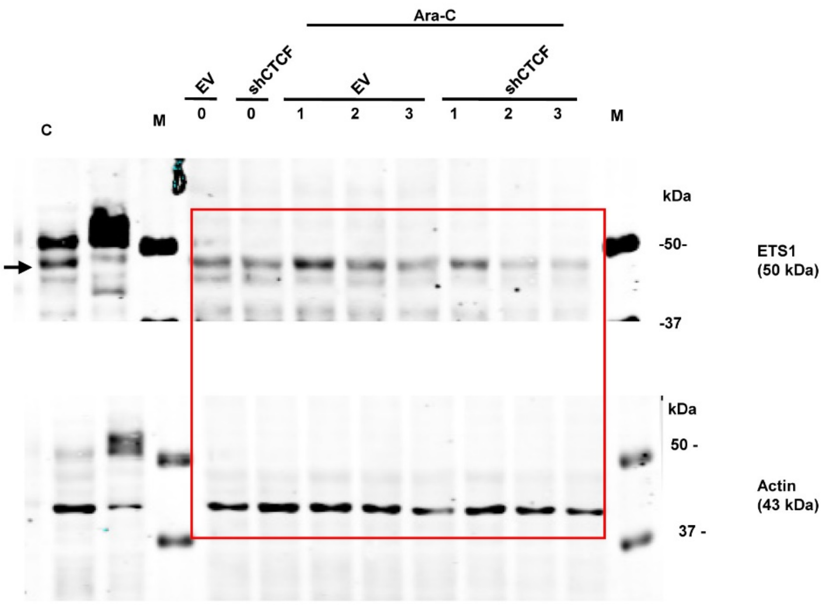

Figure 4E

ETS1 positive control (C): total lysate from Ramos cells.

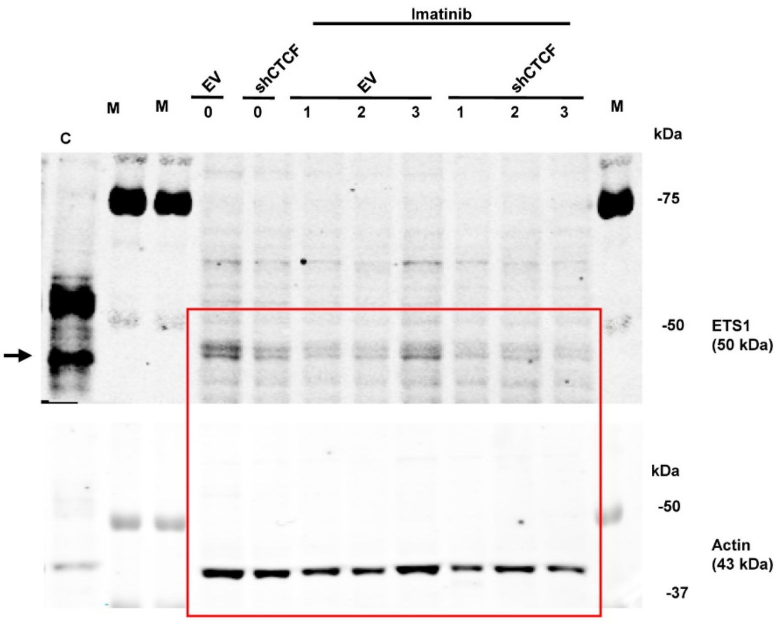

Original Western Blot images corresponding to supplementary Figures S1 and S3.

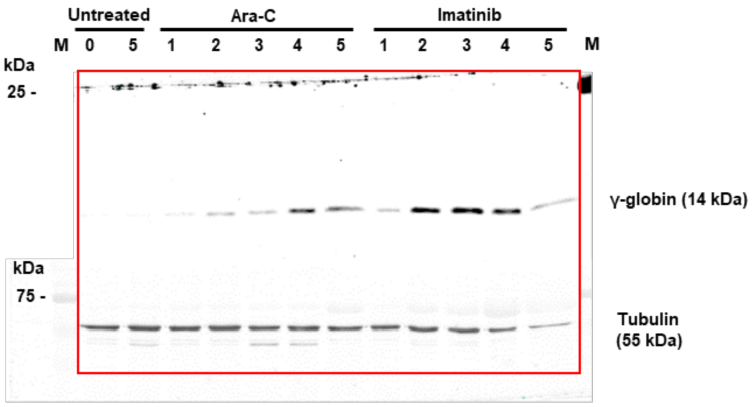

Figure S3C

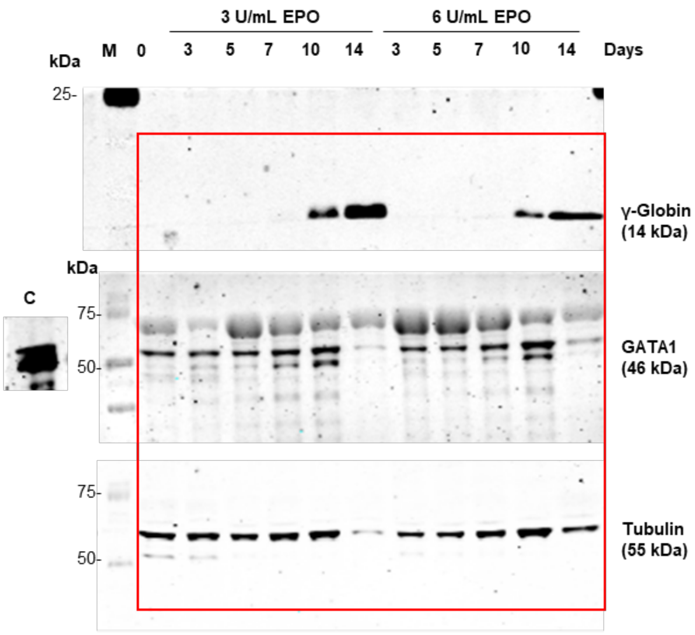

Positive control (C): 293T cells transfected with pCEFL-GATA1

Original Western Blot images corresponding to supplementary Figure S2

Figure S2B

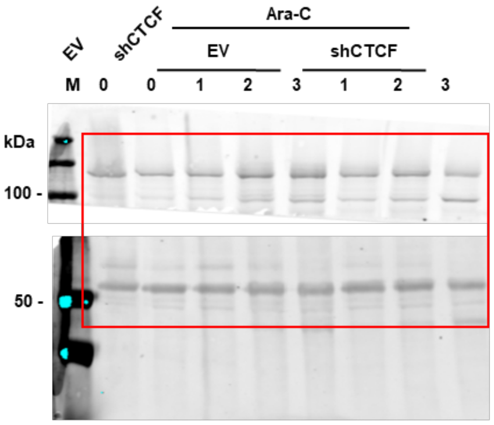

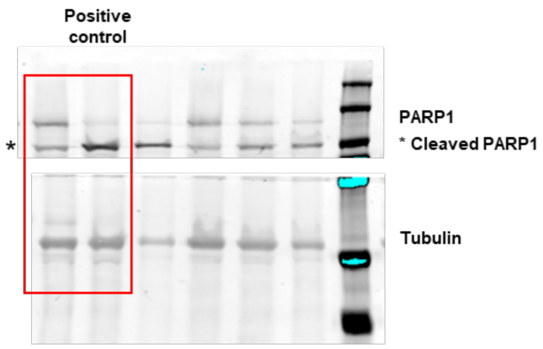

Positive control of apoptosis: K562 cells treated with high dosis (1µM) of imatinib.

Original Western Blot images corresponding to supplementary Figure S7

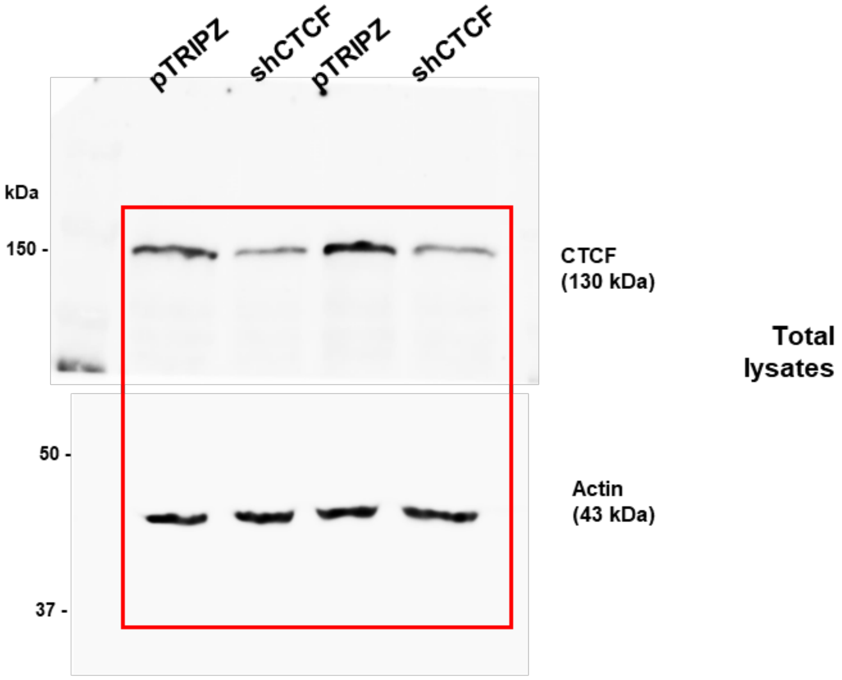

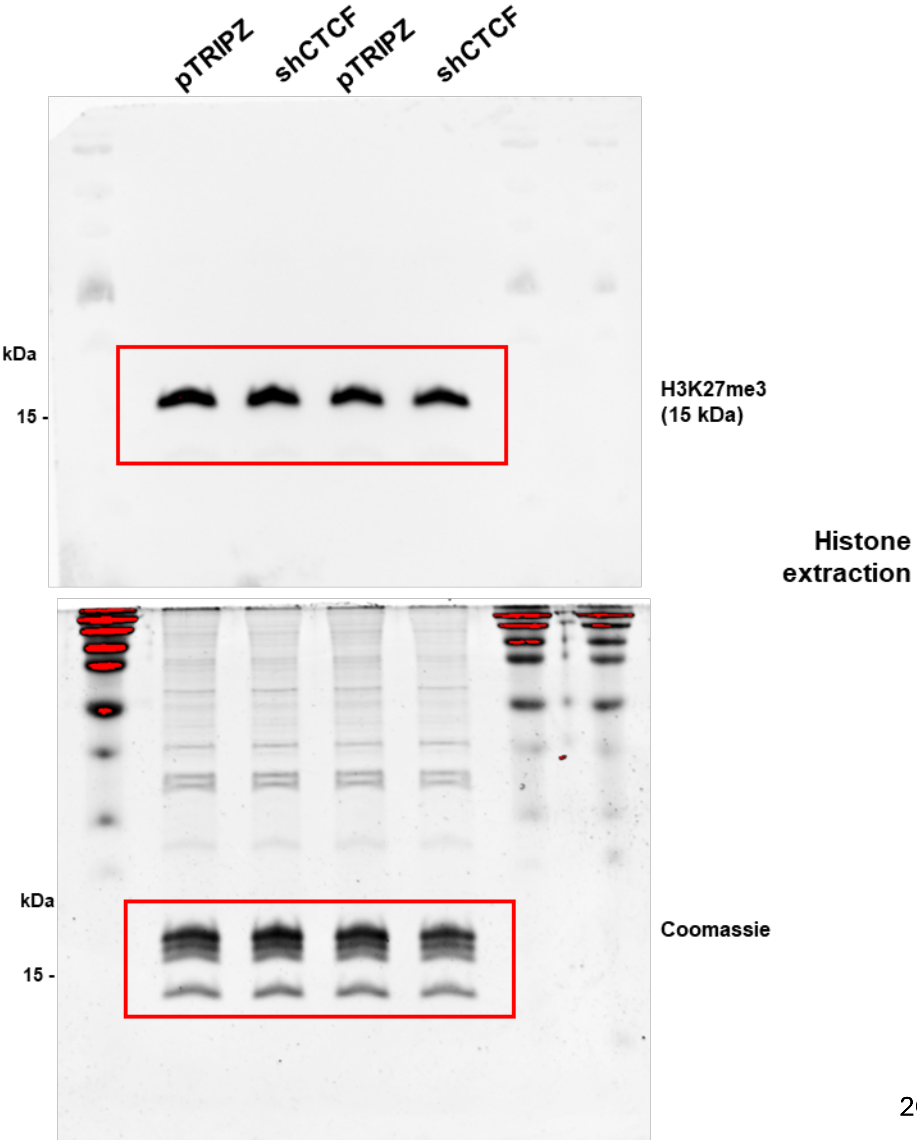

Supplement: Supplementary file 1 [file biomolecules-16-00549-s001.zip › biomolecules-4193286-supplementary.pdf]
